# Advances in understanding exosome-mediated regulation of macrophage function

**DOI:** 10.3389/fimmu.2026.1813268

**Published:** 2026-08-10

**Authors:** Yufei Qi, Xinyang Li, Jinghui Zhou, Chun Lü, Zheng Hao, Lirong Zeng

**Affiliations:** 1 College of Traditional Chinese Medicine, Tianjin University of Traditional Chinese Medicine, Tianjin, China; 2 College of Acupuncture and Tuina, Tianjin University of Traditional Chinese Medicine, Tianjin, China; 3 Tianjin Key Laboratory of Modern Chinese Medicine Theory of Innovation and Application, Tianjin University of Traditional Chinese Medicine, Tianjin, China; 4 Institute of Basic Research in Traditional Chinese Medicine Oncology, Tianjin University of Traditional Chinese Medicine, Tianjin, China

**Keywords:** exosomes, macrophage polarization, macrophages, non-coding RNAs, signaling pathways

## Abstract

Exosomes serve as crucial mediators of intercellular communication, regulating cellular functions by carrying proteins, nucleic acids, and lipids. As core components of the immune system, macrophages are closely associated with exosomes. This review focuses on three main aspects: functional outcomes, signaling pathways, and regulatory mechanisms. In terms of functional outcomes, exosomes from different origins influence macrophage polarization, activation, recruitment, and programmed cell death. Regarding signaling pathways, exosomes function primarily through two distinct modules—the conserved core pathways, represented by PI3K/Akt, STAT3, and NF-κB, and the context-dependent mechanisms, represented by MAPK and PTEN. With respect to regulatory modes, exosomes control macrophage functional differentiation via non-coding RNAs and metabolic reprogramming. In addition, this review compares the heterogeneity of exosomes derived from different sources, analyzes the mechanisms and selectivity of exosome uptake by macrophages, and outlines the key challenges and strategies for clinical translation. In summary, exosomes regulate macrophage function through multiple pathways and targets, playing critical roles in tumor biology, inflammation, and tissue repair. A deeper understanding of these mechanisms may provide new insights and therapeutic targets for exosome-based applications.

## 1 Introduction

Exosomes are extracellular vesicles with a diameter of approximately 30–200 nm, enclosed by a lipid bilayer membrane and carrying various bioactive molecules including proteins, nucleic acids, and lipids. These vesicles were first discovered and named during reticulocyte maturation (1), and were subsequently demonstrated to possess antigen-presenting function in B lymphocytes (2), thereby establishing their role as crucial mediators of intercellular communication. Exosome biogenesis begins with the formation of early endosomes through endocytosis, followed by maturation into multivesicular bodies (MVBs) and eventual release into the extracellular space (3). However, current isolation techniques cannot fully distinguish exosomes from other small extracellular vesicle (sEV) subtypes. Therefore, throughout this review, the term “exosomes” is used operationally at the experimental level. This usage is consistent with the MISEV2023 (Minimal Information for Studies of Extracellular Vesicles 2023) guidelines (4) and aligns with the nomenclature adopted by the vast majority of cited studies. Only a limited number of studies included herein have explicitly employed the term “small extracellular vesicles (sEVs)” to distinguish their isolated products from conventionally defined exosomes.

Exosomes have demonstrated considerable potential in regulating macrophage function. Macrophages belong to the mononuclear phagocyte system (MPS), originate from bone marrow hematopoietic stem cells, and are broadly distributed across various tissues and organs (5). They perform diverse functions including pathogen recognition and clearance, target cell killing, antigen presentation, and immunomodulation, thereby contributing to the maintenance of immune homeostasis (6). Macrophages can polarize into functionally distinct M1 and M2 phenotypes in response to different microenvironmental stimuli, and this plasticity serves as the cornerstone of their diverse immunological functions (7, 8). In pathological processes such as tumorigenesis, inflammation, and tissue repair, exosomes and macrophages are closely interconnected (9).

This is a narrative review that synthesizes published literature on exosome-mediated regulation of macrophage function. We conducted a comprehensive literature search in the PubMed and Web of Science databases using keywords including “exosomes”, “macrophages”, “polarization”, and “signaling pathways”. Given the narrative nature of this review, we did not follow the PRISMA (Preferred Reporting Items for Systematic Reviews and Meta-Analyses) methodology, nor did we apply formal inclusion/exclusion criteria or study quality assessment tools. Our aim is to provide a broad and integrative overview of the field, with emphasis on key mechanisms, heterogeneity considerations, and translational challenges.

## 2 Effects of exosomes on macrophages

### 2.1 Macrophage polarization

Macrophage polarization is critical for macrophage function, as distinct polarization states confer different functional properties. M1 macrophages are activated during the early phase of inflammatory responses and exhibit pro-inflammatory features. In contrast, M2 macrophages play a pivotal role in the later stages and are characterized by anti-inflammatory and tissue-repairing properties (10).

Numerous studies have demonstrated that exosomes can modulate the M1 and M2 polarization states of macrophages to improve their functions. For example, in the context of M1 polarization regulation, research has confirmed that during acute myocardial infarction, murine exosomal miR-30a derived from hypoxic cardiomyocytes inhibits autophagy in macrophages by targeting Beclin 1 (*BECN1*) and Autophagy-related 5 (*ATG5*). This inhibition mediates polarization toward the pro-inflammatory M1 phenotype, thereby triggering sustained inflammatory responses (11).

However, these findings commonly face a bottleneck: the underlying mechanisms are clearly elucidated *in vitro*, but *in vivo* evidence remains insufficient. For instance, Yan et al. (12) demonstrated that tumor-derived exosomal miR-146a inhibits M1 polarization by targeting TNF receptor-associated factor 6 (*TRAF6*) and Interleukin-1 receptor-associated kinase 1 (IRAK1), thereby enhancing tumor cell proliferation and invasion. Nevertheless, this conclusion lacks supporting data from mouse xenograft models, macrophage depletion or rescue experiments, or clinicopathological correlation analyses. Similarly, Zheng et al. (13) observed in db/db mouse models that exosomal miR-34a derived from renal tubular epithelial cells targets Peroxisome proliferator-activated receptor gamma coactivator 1-alpha (*PPARGC1A*) to promote M1 polarization and aggravate renal tubulointerstitial fibrosis. However, this finding has not yet been validated in patients with diabetic nephropathy (DN) or in independent cohorts. Zhang et al. (14) reported that cancer-associated fibroblast (CAF)-derived exosomal miR-889-3p inhibits M1 polarization by targeting Signal transducer and activator of transcription 1 (*STAT1*) to promote esophageal squamous cell carcinoma (ESCC) progression. Nonetheless, their clinical sample size was only 52 cases, and the subcutaneous xenograft model could not fully recapitulate the intercellular interactions within the esophageal orthotopic tumor microenvironment.

Currently, most studies have focused on the role of exosomes in promoting M2 polarization. In the tumor microenvironment, exosomes derived from K562 leukemia cells can drive macrophages toward M2 polarization. This process establishes an immunosuppressive microenvironment that favors leukemia cell survival (15). Exosomes derived from human hepatocellular carcinoma cell lines SMMC-7721 and HepG2 carry lipocalin 2 (LCN2) and induce M2 macrophage polarization through the Neural precursor cell expressed developmentally down-regulated protein 4-1 (NEDD4-1)/Scavenger receptor class B type I (SR-BI) axis, thereby promoting hepatocellular carcinoma progression (16). Exosomes released by paclitaxel-resistant human breast cancer SKBR-3/PR cells are enriched with miR-99b-3p. These exosomes can induce M2 macrophage polarization and amplify tumor drug resistance and metastatic phenotypes (17). Unlike tumor-derived exosomes that drive immunosuppression, exosomes derived from human primary placental mesenchymal stem cells (MSCs) carry miR-26-5p and regulate macrophage M2 polarization by targeting the PTEN/MTOR pathway. This regulation alleviates drug-induced liver injury (18). Exosomes from human primary umbilical cord MSCs deliver miR-21-5p, which targets the *SPRY2* and *MAPK1/MAPK3* (encoding ERK) pathway to promote M2 macrophage polarization, subsequently ameliorating TNBS/DSS-induced colitis in mice (19). Furthermore, exosomes from human umbilical cord MSCs overexpressing AlkB homolog 5 (*ALKBH5*) promote M2 polarization and inhibit M1 polarization through m6A demethylation of *TRAF6*, thus alleviating the progression of diabetic kidney disease (20). This provides a novel strategy for immunotherapy.

### 2.2 Macrophage activation

Macrophage activation refers to the process by which macrophages transition from a resting state to an activated state in response to stimuli such as pathogen-associated molecular patterns (PAMPs) and cytokines (21). Studies have demonstrated that exosomes are closely associated with macrophage activation. On one hand, exosomes can promote macrophage activation to facilitate immune evasion by tumor cells. For example, exosomes derived from the serum of colorectal cancer mouse models impair the immune function of CD4^+^ T cells through macrophage activation (22). Additionally, exosomes released by K562 leukemia cells, which are enriched with Peroxiredoxin 2 (PRDX2), can induce bone marrow macrophage precursors and RAW264.7 cells to differentiate into mature osteoclasts (23). On the other hand, exosomes can inhibit macrophage activation to alleviate excessive inflammatory responses. Bovine endometrial epithelial BEND cells, upon lipopolysaccharide (LPS) injury, secrete exosomes carrying low levels of miR-331. *In vitro* experiments have confirmed that exosomal miR-331 targets the Notch receptor 1 (*NOTCH1*)/Inhibitor of nuclear factor kappa-B kinase subunit alpha (*IKBKA*, encoding IKKα) pathway to suppress excessive M1-type macrophage activation, thereby alleviating inflammatory injury in endometritis (24). Furthermore, pancreatic exosomes secreted by primary injured rat pancreatic acinar cells enter the bloodstream and induce pro-inflammatory polarization of alveolar macrophages. Emodin can suppress this effect via the Peroxisome proliferator-activated receptor gamma (PPARG)/Nuclear factor kappa-light-chain-enhancer of activated B cells (*NFKB1*, encoding NF-κB) pathway, thereby improving acute lung injury secondary to pancreatitis (25). Li et al. (26) demonstrated that exosomes derived from highly metastatic cells carry high abundance of miR-193b-3p, which can be taken up by macrophages and target Mitogen-activated protein kinase kinase kinase 3 (*MAP3K3*, encoding MEKK3). This promotes the polarization of tumor-associated macrophages (TAMs) toward a pro-tumor phenotype, subsequently enhancing the invasive capacity and radio resistance of nasopharyngeal carcinoma (NPC) cells. However, this study was based solely on *in vitro* cell co-culture models and lacked *in vivo* functional validation using animal models; further confirmation through subcutaneous xenograft models is required.

Beyond tumor-derived sources, exosomes derived from immune cells also participate in the regulation of macrophage activation. Exosomes from mouse bone marrow-derived dendritic cells (DCs) pulsed with tumor antigen peptides can induce specific antitumor T-cell responses *in vivo*, providing pioneering evidence for cell-free exosome-based tumor vaccines (27). Similarly, EVs derived from immune cells are involved in modulating macrophage activation. For instance, extracellular vesicles (EVs) released by activated human and mouse CD4^+^ T cells, which carry interferon-gamma (IFNγ) on their surface, can sensitize the Stimulator of interferon genes (STING) pathway in macrophages. This promotes the release of type I interferons (IFNs) and C-X-C motif chemokine ligand 10 (CXCL-10), synergistically enhancing STING agonist-mediated antitumor immunity (28).

### 2.3 Macrophage recruitment

Macrophage recruitment refers to the process by which the body attracts macrophages from the circulation or surrounding tissues to specific sites through a series of signaling mechanisms in response to pathogen invasion or tissue injury, thereby playing a critical role in immune responses and tissue repair (29). In terms of pro-recruitment effects, exosomes derived from various pathological sources can enhance macrophage recruitment and inflammatory infiltration. For example, exosomes secreted by SiO_2_-stimulated RAW264.7 and THP-1 cells carry High mobility group box 3 (HMGB3) and promote monocyte-macrophage recruitment through the Signal transducer and activator of transcription 3 (STAT3)/Mitogen-activated protein kinase (MAPK)/NF-κB)/C-C motif chemokine receptor 2 (CCR2) pathway, thereby exacerbating pulmonary inflammation. Related *in vivo* validation was performed using a C57BL/6 silicosis mouse model (30). Additionally, exosomes released by H1N1-infected human lung epithelial A549 cells can synergize with the autophagy pathway to jointly promote macrophage recruitment and M1-type polarization during influenza A virus infection (31).

In contrast to the above pro-recruitment effects, some exosomes exhibit atypical inhibitory effects on macrophage recruitment. Zhang et al. found that the mechanism by which antler stem cell-derived exosomes alleviate pulmonary fibrosis does not directly involve inhibition of M2 polarization. Instead, these exosomes reduce the secretion of C-C motif chemokine ligand 7 (CCL7) by fibroblasts, which in turn decreases monocyte-macrophage recruitment and consequently reduces the local M2 macrophage population (32). This finding suggests that exosomes may operate through non-canonical regulatory pathways. However, the unique origin of antler stem cells raises questions regarding the generalizability of these effects, and there is a certain disconnect between the *in vitro* and *in vivo* results. Collectively, these studies indicate that the regulation of macrophage recruitment by exosomes is bidirectional, encompassing both pro-recruitment pathways and atypical inhibitory mechanisms.

### 2.4 Macrophage programmed cell death

Programmed cell death refers to the active and orderly process by which cells undergo death under physiological or pathological conditions, encompassing apoptosis, pyroptosis, autophagy, and other forms. This process is of great significance for maintaining immune homeostasis and various other physiological functions (33). In terms of apoptosis regulation, exosomes can influence macrophage fate by delivering long non-coding RNAs (lncRNAs). In silicosis research, exosomes secreted by silica-stimulated macrophages carry the lncRNA MSTRG.43085.16, which promotes macrophage apoptosis through upregulation of Poly(ADP-ribose) polymerase 1 (PARP1) (34). However, this lncRNA has no matching record in genomic databases, and direct binding between MSTRG.43085.16 and PARP1 lacks experimental validation; thus, its function requires further confirmation.

Furthermore, exosomes containing miR-29a-3p, secreted by macrophages differentiated from THP-1 monocytes, can upregulate the expression of B7 homolog 3 (B7-H3). B7-H3 subsequently activates the NF-κB pathway to upregulate Hypoxia-inducible factor 1-alpha (HIF-1α), thereby enhancing the anti-apoptotic capacity of macrophages under hypoxic conditions (35). Meanwhile, the exosomal lncRNA AU020206 secreted by mouse primary bone marrow-derived mesenchymal stem cells alleviates atherosclerosis by inhibiting macrophage pyroptosis (36). In mouse models of spinal cord injury and BV2 microglial cell experiments, exosomal let-7b-5p derived from neural stem cells differentiated from induced pluripotent stem cells (iPSCs) was shown to inhibit microglial/macrophage pyroptosis and promote functional recovery of motor function after spinal cord injury (37).

**Figure 1:**
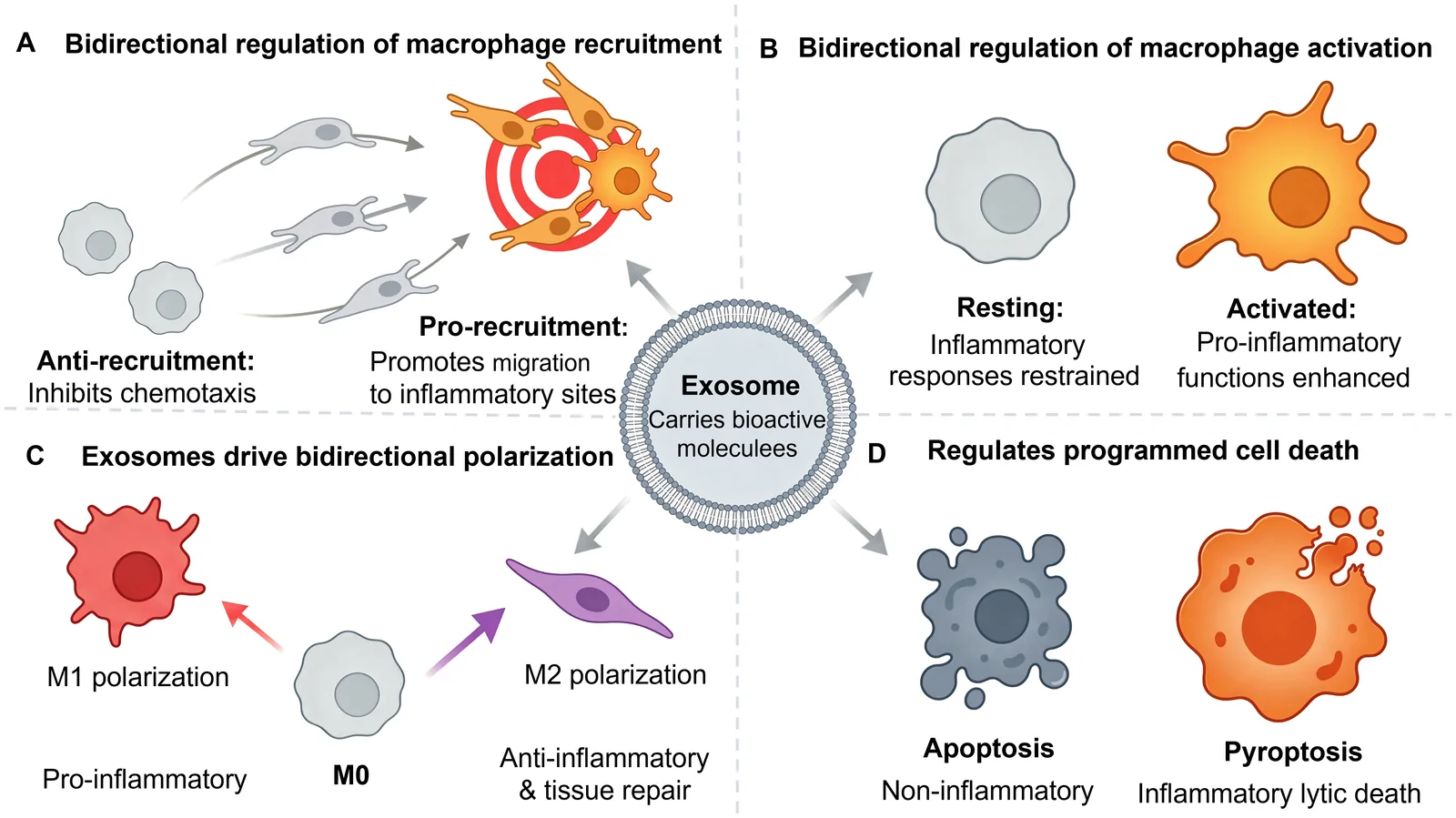
Schematic overview of exosome-mediated regulation of macrophage functions. Schematic overview of exosome-mediated regulation of macrophage functions. The figure illustrates four major functional outcomes of macrophage regulation by exosomes: polarization, activation, programmed cell death, and recruitment. Exosomes carry bioactive molecules and regulate macrophage functions from four dimensions: **(A)** regulation of macrophage recruitment, which can either inhibit chemotaxis to achieve anti-recruitment effects or promote macrophage migration toward inflammatory sites to exert pro-recruitment effects; **(B)** regulation of macrophage activation status, which can either maintain macrophage quiescence and constrain inflammatory responses or enhance pro-inflammatory functions to activate macrophages; **(C)** induction of macrophage polarization, which can drive resting M0 macrophages toward pro-inflammatory M1 polarization or toward anti-inflammatory and tissue-repairing M2 polarization; **(D)** regulation of macrophage programmed cell death, which can mediate non-inflammatory apoptosis or induce pyroptosis accompanied by inflammatory effects.

In summary, exosomes exert multidimensional regulation of macrophage functions by delivering bioactive molecules (**Figure 1**). In terms of polarization, exosomes can bidirectionally regulate M1 and M2 phenotypes, influencing inflammatory progression and the tumor microenvironment. Regarding activation, tumor-derived exosomes can enhance macrophage activation to promote tumor progression; conversely, exosomes can exert anti-inflammatory effects by inhibiting excessive macrophage activation. With respect to recruitment, exosomes can bidirectionally modulate macrophage recruitment, thereby alleviating pulmonary inflammation and fibrosis. Additionally, exosomes can attenuate macrophage apoptosis and pyroptosis.

## 3 Signaling pathways through which exosomes regulate macrophages

The regulation of macrophage polarization, activation, recruitment, and programmed cell death by exosomes is fundamentally mediated by signaling cascades triggered upon entry of exosomal cargo into macrophages. Currently, it is established that exosomes primarily exert their functional reprogramming of macrophages through classical pathways, including Phosphoinositide 3-kinase (PI3K)/Protein kinase B (AKT, also known as PKB), STAT3, NF-κB, MAPK, and PTEN. A careful examination of the mechanisms by which these pathways regulate macrophage polarization reveals that some pathways, once activated or inhibited, drive phenotypic transformation in a relatively consistent direction. In contrast, the ultimate outcomes of other pathways depend on the inflammatory or reparative context of the cellular environment. Based on this distinction, we categorize these pathways into two major groups for discussion.

### 3.1 Conservative core pathways: PI3K/AKT, STAT3, and NF-κB

Activation or inhibition of the PI3K/AKT, STAT3, and NF-κB pathways generally directs polarized phenotypic shifts in a highly reproducible manner across different disease contexts.

The PI3K/AKT pathway is a critical signaling network that regulates cell proliferation, survival, and metabolism (38). Zheng et al. demonstrated that exosomes secreted by mouse primary adipose-derived MSCs after LPS pretreatment deliver miR-150-5p to target Insulin receptor substrate 1 (*IRS1*), thereby inhibiting the PI3K/AKT/mTOR pathway. This promotes M2 macrophage polarization and alleviates inflammatory injury in sepsis (39). Exosomes secreted by M2 macrophages differentiated from IL-4-induced mouse RAW264.7 cells promote M2 macrophage polarization via the PI3K/AKT pathway, thereby improving diabetic wound healing in mice (40). Furthermore, in tumor-related diseases, exosomes derived from the human esophageal squamous cell carcinoma cell line EC109 deliver Hyaluronidase 1 (HYAL1) to THP-1-derived macrophages. Through the HYAL1/Aurora kinase B (AURKB) axis, these exosomes activate the PI3K/AKT pathway to mediate M2 macrophage polarization, ultimately promoting esophageal squamous cell carcinoma cell proliferation and invasion (41). *In vitro*, exosomes released by human esophageal epithelial cells, Barrett’s esophageal cells, and esophageal adenocarcinoma tumor cells following stimulation with acidic bile salts activate the PI3K/AKT signaling pathway to mediate M2 macrophage polarization, ultimately driving esophageal adenocarcinoma cell proliferation (42). Exosomes secreted by degenerated nucleus pulposus cells carry high abundance of miR-27a-3p, which targets and inhibits PPARγ in macrophages. This suppresses the PI3K/AKT pathway and activates the NF-κB signaling pathway, inducing pro-inflammatory M1 polarization of macrophages and forming a vicious inflammatory cycle (43). The vast majority of the above evidence indicates that activation or inhibition of this pathway points toward M2 polarization across multiple disease models, which is consistent with its role as a core switch for metabolic reprogramming. It should be noted that when exosomal cargo simultaneously and directly activates NF-κB, this pathway can also drive M1 polarization; the regulatory direction depends on the synergistic status of associated transcriptional signals.

The STAT3 signaling pathway is a transcriptional regulatory network that governs cell proliferation, differentiation, and immune responses, and plays an extremely important role in macrophage polarization (44). In terms of STAT3 pathway activation, for example, exosomes from mouse GL261 glioma stem cells promote glioma progression through the Nuclear paraspeckle assembly transcript 1 (*NEAT1*)/miR-125a/STAT3 pathway (45). Exosomal circular RNA circ_001264 from human THP-1/MOLM13 leukemia cells competitively binds miR-502-5p, upregulating Raf-1 proto-oncogene, serine/threonine kinase (*RAF1*) to activate the p38-STAT3 pathway, thereby mediating leukemia immune evasion (46). In experiments using NSG mice and human colorectal cancer tissues, exosomes derived from HCT116 and SW620 cells act on THP-1 macrophages to induce M2 polarization through the IL-10/STAT3 pathway, driving colorectal cancer metastasis (47). However, the clinical sample size in this study was only 28 cases from a single center, and the clinical value of circPOLQ as a biomarker or therapeutic target requires validation in large-scale prospective cohort studies.

Interestingly, exosomes derived from human umbilical cord MSCs can exert anti-inflammatory effects by inhibiting both NF-κB and STAT3 pathway activation, similarly suppressing M1 polarization while promoting M2 polarization (48). Exosomes secreted by cigarette smoke-stimulated human bronchial epithelial cells carry miR-221-3p and transfer it to macrophages. Through the Suppressor of cytokine signaling 3 (*SOCS3*)/STAT3 signaling pathway, these exosomes promote macrophage polarization toward the pro-inflammatory M1 phenotype, continuously driving pulmonary inflammation progression in COPD (49). The above examples demonstrate that the polarization-directing role of STAT3 is not fixed but strictly depends on which upstream signals activate it.

The NF-κB signaling pathway serves as a major regulator of inflammatory responses, immune responses, and cell survival (50). Exosomes derived from human adipose-derived MSCs and mouse primary bone marrow-derived MSCs promote M2 macrophage polarization by inhibiting the NF-κB pathway, thereby alleviating inflammatory responses (51) and myocardial infarction (52), respectively. During tissue repair, exosomes from LPS-pretreated rat primary bone marrow-derived MSCs promote M2 macrophage polarization through the NF-κB/NLRP3 pathway, thereby prolonging allograft survival (53). HMGB1 in exosomes secreted by human gastric cancer cells MGC-803 and OCUM-1 promotes M2 macrophage polarization by blocking NF-κB signaling, thereby remodeling the immunosuppressive tumor microenvironment (54). After being taken up by macrophages, CLP-exo induces NF-κB pathway activation, driving M2 macrophage polarization, prolonging inflammatory cell survival, and amplifying sepsis-related inflammatory responses (55). The above evidence indicates that NF-κB regulation of polarization generally follows the pattern that activation drives M1 polarization while inhibition directs toward M2 polarization.

### 3.2 Context-dependent special mechanisms: MAPK and PTEN

Unlike the core hubs discussed above, MAPK and PTEN primarily function in signaling networks as upstream stress sensors or bypass regulators of core pathways. Their direction toward promoting M1 or M2 polarization depends on the inflammatory or reparative state of the tissue microenvironment.

The MAPK signaling pathway is a core signaling network that regulates cell proliferation, death, differentiation, inflammation, and immune responses (56). In inflammatory diseases, MAPK pathway activation often drives macrophages toward the M1 phenotype, further promoting inflammation. For example, exosomes derived from the plasma of patients with coronary heart disease are enriched with lnc-MRGPRF-6:1, which promotes M1 polarization of human primary monocyte-derived macrophages and THP-1 cells through the Toll-like receptor 4 (*TLR4*)/Myeloid differentiation primary response 88 (*MYD88*)/MAPK pathway, thereby amplifying inflammatory responses in coronary atherosclerosis (57). Exosomal miR-26b-3p derived from primary hypertrophic mesenteric adipose tissue in patients with Crohn’s disease drives macrophages toward M1 polarization and amplifies intestinal anastomotic inflammation through the Tripartite motif-containing 33 (*TRIM33*)/p38-MAPK pathway (58). However, the validation of *TRIM33* as a target relied solely on dual-luciferase reporter assays, lacking direct molecular interaction evidence, and the sample size was relatively small.

In tissue repair contexts, however, the role of MAPK is distinctly different. For example, exosomes derived from human primary adipose-derived MSCs (hAdMSCs) promote M2 macrophage polarization by targeting and inhibiting the p38 MAPK/NF-κB signaling pathway, thereby repairing facial nerve injury in rats (59). Additionally, exosomes from rat primary bone marrow-derived MSCs pretreated with TGF-β1 deliver miR-135b to target Mitogen-activated protein kinase 6 (*MAPK6*), inducing synovial macrophage M2 polarization and alleviating cartilage damage in osteoarthritis (60). The fact that the same pathway promotes M1 polarization in inflammation but M2 polarization in repair suggests that the ultimate effect is not determined by the pathway alone, but rather by synergistic signals from the microenvironment.

The PTEN signaling pathway is a critical molecular hub that regulates cell proliferation, metabolism, and tumorigenesis (61), and its functional direction similarly varies with the context. In the tumor microenvironment, exosomes drive M2 macrophage polarization by inhibiting the PTEN pathway, thereby exacerbating tumor progression and metastasis. For example, exosomes derived from colorectal cancer HCT116 cells deliver miR-203a-3p to macrophages, which inhibits PTEN and activates the PI3K/AKT pathway *in vitro* to induce M2 polarization, thereby promoting colorectal cancer invasion and metastasis (62). Exosomes secreted by human NSCLC cell lines A549 and PC9 carry circFARSA and induce M2 macrophage polarization through the PTEN/PI3K/AKT pathway, promoting non-small cell lung cancer progression (63). In metabolic and inflammatory diseases, exosomal regulation of the PTEN signaling pathway exhibits functional diversity. Exosomal miR-301a-3p derived from ectopic endometrial tissue of patients with endometriosis promotes M2 polarization of THP-1-derived macrophages through the PTEN-PI3K pathway, mediating local inflammation and fibrosis progression in ectopic endometrium (64). In contrast, in diabetic wound healing, exosomes secreted by human primary bone marrow-derived MSCs pretreated with melatonin remodel macrophage polarization by upregulating PTEN and inhibiting AKT phosphorylation. This increases the M2/M1 ratio to alleviate local inflammation, ultimately accelerating skin wound repair in STZ-induced diabetic rats (65). In tumors, PTEN inhibition promotes M2 polarization, whereas in wounds, PTEN upregulation similarly promotes M2 polarization. This indicates that the ultimate effect of PTEN is determined by the regulatory demands of the specific pathological context.

In summary, exosomal regulation of macrophage polarization is not determined by the activation or inhibition of any single pathway in isolation, but rather results from the coordinated action of the two major signaling modules described above. This interplay and signal integration among pathways enables exosomes to achieve differential reprogramming of macrophage functions across various pathological contexts (**Table 1**).

**Table 1:** Exosome-mediated regulation of macrophage signaling pathways.

Exosome source	Exosomes cargo	Pathway-target	Macrophage regulation	Disease	References
PI3K/Akt					
Adipose MSCs	miR-150-5p	PI3K/Akt/mTOR	M2 polarization	Sepsis	(39)
M2 macrophages	–	PI3K/Akt		Diabetic wound	(40)
ESCC-Macrophage co-culture	HYAL1	PI3K/AKT		Esophageal squamous cell carcinoma	(41)
Serum	EGFR	PI3K-AKT		Esophageal adenocarcinoma	(42)
Colorectal cancer tumor cells	miR-203a-3p	PI3K/Akt		Colorectal cancer liver metastasis	(62)
Adipose MSCs	–	PI3K/AKT/mTOR		Glioblastoma	(66)
Umbilical cord MSCs	miR-486-5p	PI3K/Akt-PIK3R1		Diabetic Nephropathy	(67)
Nucleus pulposus cells	miR-27a-3p	PPARγ/NFκB/PI3K/AKT	M1 polarization	Intervertebral disc degeneration	(43)
STAT3					
Colorectal cancer cells	circRNA POLQ	IL-10/STAT3	M2 polarization	Colorectal cancer	(47)
Acute myeloid leukemia cells	circRNA-001264	p38-STAT3		Acute myeloid leukemia	(46)
Human umbilical cord MSCs	–	NF-κB p65,STAT3		Inflammatory diseases	(48)
Hepatocellular carcinoma cells	PSMA5	JAK2/STAT3		Hepatocellular carcinoma	(68)
Gastric cancer cells	miR-541-5p	DUSP3/JAK2/STAT3		Gastric cancer	(69)
Bronchial epithelial cells	miR-221-3p	STAT3	M1 polarization	COPD	(49)
NF-κB					
Adipose stem cells	Icariin	*TLR4*/*Myd88*/NF-κB	M2 polarization	Inflammatory diseases	(51)
Bone marrow MSCs	CISH	NF-κB		Myocardial infarction	(52)
Bone marrow MSCs	–	NF-κB/NLRP3/procaspase-1/IL-1β		Allograft survival	(53)
Gastric cancer cells	HMGB1	NF-κB		Gastric cancer	(54)
Sepsis serum	MIR17HG	NF-κB/miR-17-92a-1	Macrophage activation	Sepsis	(55)
MAPK					
Adipose MSCs	–	P38 MAPK/NF-κB	M2 polarization	Facial nerve injury	(59)
Adipose MSCs	miR-135b	*MAPK6*		Cartilage injury	(60)
Plasma	LncRNA MRGPRF-6:1	*TLR4*-*MyD88*-MAPK	M1 polarization	Coronary artery disease	(57)
Macrophages	miR-155-5p	MSK1/p38-MAPK		Acute lung injury	(70)
Dental pulp stem cells	–	ROS-MAPK-NFκB P65		Spinal cord injury	(71)
PTEN					
Non-Small cell lung cancer cells	circFARSA	PTEN/PI3K/AKT	M2 polarization	Non-Small cell lung cancer metastasis	(63)
Serum	miR-301a-3p	TEN-PI3K		Endometriosis	(64)
Umbilical cord MSCs	–	PTEN/AKT		Diabetic wound	(65)
Esophageal squamous cell carcinoma cells	miR-301a-3p	PTEN/PI3K/AKT		Esophageal squamous cell carcinoma	(72)
Breast cancer cells	miR-222	PTEN/Akt		Breast cancer	(73)
Fibroblasts	miR-320a	PTEN/PI3K γ		Pancreatic cancer	(74)

## 4 Pathways through which exosomes regulate macrophages

### 4.1 Exosomes regulate macrophages via non-coding RNAs

This section focuses on how non-coding RNAs (ncRNAs) carried by exosomes achieve the aforementioned regulatory effects by targeting key molecules in signaling pathways. Exosomal miRNAs, lncRNAs, circRNAs, and other ncRNAs can dynamically regulate macrophage functions by targeting key molecules in signaling pathways (75). Among these, lncRNAs and circRNAs are particularly critical in shaping the tumor immune microenvironment, inflammatory responses, and tissue repair (**Tables 2**, **3**).

**Table 2:** Exosome-mediated regulation of macrophages via lncRNA.

Exosome source	Exosomal lncRNAs	Pathway-target	Macrophage regulation	References
Glioma stem cells	lncRNA *NEAT1*	miR-125a/STAT3	M2 polarization	(45)
Laryngeal squamous cell carcinoma	AC068768.1	miR-139-5p/*NOTCH1*/STAT3		(77)
MKN45 cells	*MIR4435-2HG*	Jagged1/Notch, JAK1/STAT3		(78)
Adipose MSCs	DLEU2	miR-106a-5p/LXN		(82)
Hypoxic endothelial cells	lncRNA HAR1B	BHLHE23/*KLF4*		(83)
Hepatocytes	*SLC16A1-AS1*	–		(87)
Breast cancer cells	HAGLROS	miR-135b-3p/COL10A1		(88)
Fibroblasts	lncRNA 01833	miR-335-5p-VAPA		(89)
Renal cell carcinoma	lncRNA ARSR	STAT3		(90)
M2 Macrophages	AK083884	*PKM2*/HIF-1α	Macrophage glycolysis	(85)
Bone marrow stem cells	AU020206	CEBPB/NLRP3	Macrophage pyroptosis	(36)
Airway epithelial cells	*MEG3*	SPI1/METTL3/TREM-1	M1 polarization and pyroptosis	(86)
Alveolar macrophage line NR8383 co-cultured with polymorphonuclear neutrophils	HCG18	–	M1 polarization	(91)
Antisense oligonucleotide-loaded exosomes	Lncenc1	–	Macrophage activation	(92)

**Table 3:** Exosome-mediated regulation of macrophages via circRNA.

Exosome source	Exosomal circRNAs	Pathway-target	Macrophage regulation	References
Non-small cell lung cancer	circRNA PLEKHM1	–	M2 polarization	(80)
Non-small cell lung cancer	circRNA PACRGL	Hippo		(79)
Ginsenoside RG1-induced MSCs	circRNA NOTCH1	–		(84)
Macrophage and lung adenocarcinoma cell co-culture	circRNA 0001715	miR-205-5p/TREM2		(93)
Adipose MSCs	circRNA Snhg11	miR-144-3p/HIF-1α		(94)
mSCAR-encapsulated exosomes	mSCAR	–		(95)
Melanoma	circRNA PIK3R3	miR-872-3p/*IRF7*	M1 polarization	(81)

In the tumor microenvironment, exosomes induce M2 polarization through *NEAT1* thereby promoting tumor immune evasion and metastasis (76). Exosomes derived from laryngeal squamous cell carcinoma AMC-HN8 and TU686 cells carry AC068768.1 and induce M2 polarization of THP-1 macrophages, promoting tumor metastasis (77). Exosomes secreted by breast cancer MDA-MB-231 and Hs578T cells carry MIR4435-2HG, which activates the M2 polarization program in macrophages and establishes a pro-tumor microenvironment (78). Similarly, exosomal circRNAs also play important roles in tumor-associated macrophage polarization. For example, exosomal circPLEKHM1 and circPACRGL activate the Hippo signaling pathway to induce M2 polarization (79). Exosomes secreted by hypoxic human non-small cell lung cancer A549, PC9, and H1299 cells carry circPLEKHM1, which subsequently induces M2 polarization of macrophages (80). Exosomes released by fractionated radiotherapy-treated mouse melanoma B16F10 cells contain circPIK3R3, which drives macrophages toward M1 polarization through the miR-872-3p/Interferon regulatory factor 7 (*IRF7*) signaling axis (81).

In terms of anti-inflammatory effects, exosomal lncRNA DLEU2, lncHAR1B, and circNOTCH1 all induce M2 polarization, promoting anti-inflammatory responses and tissue repair (82–84). Hypoxic exosomes derived from human primary umbilical vein endothelial cells (HUVECs) deliver lncHAR1B, which induces M2 macrophage polarization by upregulating Krüppel-like factor 4 (*KLF4*), thereby alleviating inflammatory responses and promoting diabetic wound healing (83). In addition, exosomes secreted by mouse primary M2 bone marrow-derived macrophages carry lncRNA AK083884, which inhibits excessive glycolytic reprogramming of macrophages by regulating the Pyruvate kinase M2 (*PKM2*)/HIF-1α axis, thereby alleviating Coxsackievirus B3 (CVB3)-induced viral myocarditis in mice (85). Using chronic obstructive pulmonary disease as a model, Wang et al. demonstrated that exosomes derived from cigarette smoke extract-treated mouse airway epithelial cells carry high abundance of lncRNA *MEG3*. This lncRNA recruits the transcription factor SPI1 to activate transcription of the m6A methyltransferase METTL3, which subsequently enhances m6A methylation modification of Triggering receptor expressed on myeloid cells 1 (*TREM1*) mRNA and upregulates its protein expression. This ultimately drives M1 macrophage polarization and pyroptosis. However, this regulatory cascade involves as many as six consecutive steps, each of which is positively regulated, raising questions about its robustness in the complex *in vivo* environment. To date, this mechanism has only been validated within the same research group, with no independent replication studies reported; its generalizability and pathophysiological significance require extensive further confirmation (86).

**Table 4** summarizes representative molecules from the above three categories of ncRNAs and their corresponding signaling pathways, to systematically illustrate the regulatory correlations between exosomal non-coding RNAs (ncRNAs) and the signaling pathways they modulate.

**Table 4:** Correspondence between exosomal ncRNAs and downstream signaling pathways in macrophage regulation.

ncRNA molecule	Exosome source	Targeted signaling pathway/mechanism	Macrophage regulation	References
NF-κB				
miR-1246	Adipose stem cells	*TRAF6*/NF-κB (inhibition)	Promotes M2 polarization	(96)
miR-24-3p	Human umbilical cord MSCs	STING/NF-κB/JNK (inhibition)	Inhibits M1, promotes M2	(97)
miR-216a-5p	Serum exosomes (liver IRI)	*TLR4*/NF-κB, PI3K/AKT	Promotes M2 polarization	(98)
STAT3				
HEIH	Hepatocellular carcinoma cells	miR-98-5p/STAT3 (activation)	Promotes M2 polarization	(76)
circRNA ATG7	Urine-derived stem cells	miR-4500/SOCS1/STAT3	Inhibits M1, promotes M2	(99)
PI3K/AKT				
circ-CBLB	RA-FLSs	TLR3/TRAF3 (inhibition)	Inhibits M1 polarization	(100)
miR-125b-5p	Pancreatic acinar cells	IGF2/PI3K/AKT	Inhibits M2, promotes M1	(101)
other signaling pathways				
miR-26-5p	Human placental MSCs	PTEN/mTOR (activation)	Promotes M2 polarization	(18)
SLC12A2-DT	Hepatocellular carcinoma cells	Wnt/GSK3β/β-catenin (activation)		(102)
H19	Cholangiocytes under cholestatic conditions	CCL-2/CCR-2 (activation)	Promotes M1 polarization	(103)
miR-146a-5p	LPS-activated hepatic stellate cells	KLF-4/JNK (activation)		(104)
Rmrp	Type II alveolar epithelial cells	ZFP36/PFKFB3 (glycolysis inhibition)	Macrophage immunosuppression	(105)

### 4.2 Exosomal regulation of macrophage metabolic reprogramming

Macrophage metabolic reprogramming refers to the process by which macrophages adapt to microenvironmental stimuli and promote their differentiation through glycolysis, oxidative phosphorylation, lipid metabolism, and other metabolic pathways (106). This reprogramming is a core driver of macrophage phenotype and function (107). As key mediators of intercellular communication, exosomes can regulate macrophage metabolic pathways, thereby influencing their immune phenotype and function.

#### 4.2.1 Glycolysis pathway

The glycolysis pathway participates in numerous immune responses and is essential for immune cell function (108). It also serves as the primary energy source for M1 macrophage polarization, and its excessive activation promotes inflammatory responses (109).

In terms of inhibiting macrophage glycolysis, exosomes derived from human primary adipose-derived stem cells (ADSCs) loaded with icariin have been shown to inhibit glycolytic reprogramming and promote M2 polarization in RAW264.7 macrophages and collagen-induced arthritis (CIA) rat models, thereby alleviating joint inflammation (110). Exosomes inhibit the glycolysis pathway to promote M2 polarization, thereby accelerating bone defect repair (111). Exosomes derived from Rg1-pretreated human primary ADSCs deliver miR-574-3p to block the Rat sarcoma virus (*RAS*) pathway and inhibit glycolysis in mouse RAW264.7 macrophages, thereby regulating the macrophage polarization balance and alleviating DSS-induced colitis in mice (112).

In terms of activating macrophage glycolysis, exosomes derived from hepatocellular carcinoma Hep3B cells deliver lncRNA miR4458HG, which upregulates Arginase 1 (*ARG1*) expression through the competing endogenous RNA (ceRNA) pathway, inducing M2 polarization of human monocyte-derived macrophages (113). In osteoarthritis, exosomes secreted by inflammatory cytokine-stimulated primary mouse/human fibroblast-like synoviocytes (FLSs) are internalized by macrophages, where they upregulate Hypoxia-inducible factor 1-alpha (*HIF1A*) to remodel macrophage immunometabolism and promote glycolysis, ultimately inducing macrophage polarization toward the pro-inflammatory M1 phenotype (114). Exosomes isolated from the plasma of patients with secondary hemophagocytic lymphohistiocytosis (sHLH) carry circMETTL3, and the METTL3-156aa peptide encoded by this circRNA binds to Lactate dehydrogenase A (LDHA) to promote glycolysis in human THP-1 monocyte-derived macrophages. The glycolytic product lactate upregulates the splicing factor Serine/arginine-rich splicing factor 10 (SRSF10) through histone lactylation, which in turn promotes circMETTL3 generation in a feedback loop. This forms a positive feedback circuit that continuously amplifies M1 macrophage polarization (115).

#### 4.2.2 Lipid metabolism pathway

The lipid metabolism pathway, which consists of fatty acid oxidation and lipid synthesis, is a critical basis for maintaining the M2 macrophage phenotype (116). Exosomes can promote M2 polarization by regulating lipid metabolic enzymes. For example, glioma-derived exosomes upregulate ATP-citrate lyase (ACLY) and Elongation of very long chain fatty acids protein 6 (ELOVL6), promoting lipid accumulation and fatty acid oxidation (117), thereby driving M2 polarization and facilitating tumor immune evasion. Jin et al. reported that exosomes derived from esophageal squamous cell carcinoma carry N-acetyltransferase 10 (NAT10), which enhances the stability of Fatty acid synthase (*FASN*) mRNA through ac4C modification, thereby promoting lipid metabolic reprogramming in macrophages and driving M2 polarization. However, the transcriptome-wide distribution of ac4C modifications in macrophages and the substrate specificity of NAT10 have not yet been validated by independent studies (118).

These studies indicate that exosomes regulate macrophage lipid metabolism through multiple mechanisms—including modulation of the balance between lipid synthesis and fatty acid oxidation, post-translational modification of metabolic enzymes, and miRNA-mediated reprogramming of metabolic pathways—ultimately converging to drive macrophage phenotypic transformation.

#### 4.2.3 Cross-regulation between metabolism and inflammatory signals

Exosome-mediated metabolic reprogramming of macrophages is closely intertwined with classical inflammatory signaling pathways, and these two processes mutually influence each other.

Tumor-derived exosomes drive macrophages toward an immunosuppressive phenotype and upregulate programmed death-ligand 1 (PD-L1) expression through NF-κB-dependent metabolic reprogramming that favors glycolysis (119). However, the “non-classical M1” paradigm proposed in this study remains a preliminary concept, and the generalizability of the lactate-NF-κB-PD-L1 positive feedback loop requires independent validation in additional cancer types. Moreover, the clinical sample size was relatively small, and the causal relationship between YKT6 v-SNARE homolog (*YKT6*) and PD-L1 still requires functional experimental confirmation. The glycolytic product lactate is not merely an intermediate metabolite of energy metabolism; it can also directly regulate the transcription of inflammation-related genes in macrophages through histone lactylation (120).

Furthermore, exosomes can simultaneously regulate metabolic status and inflammatory signals by delivering metabolism-related enzymes or proteins. Exosomal Enolase 2 (ENO2) derived from diffuse large B-cell lymphoma enhances glycolysis and drives M2 polarization through activation of the Glycogen synthase kinase 3 beta (*GSK3B*)/Catenin beta 1 (*CTNNB1*, encoding β-catenin)/MYC proto-oncogene (*MYC*, encoding c-Myc) pathway (121). After being taken up by macrophages, exosomal Fatty acid-binding protein 5 (FABP5) derived from hepatocellular carcinoma promotes lipid accumulation by activating the PPARγ signaling pathway, while simultaneously inhibiting fatty acid oxidation by suppressing the PPARα signaling pathway. This dual regulation of lipid metabolic reprogramming drives macrophages toward M2 polarization and promotes hepatocellular carcinoma progression (122). Exosomal miR-4488 derived from hypoxic tumors enhances fatty acid oxidation by targeting Reticulon 3 (*RTN3*)/FABP5 axis, and activates the PI3K/AKT/mTOR pathway to drive M2 polarization (123). In addition, exosomes can influence inflammatory responses by modulating protein acetylation status in macrophages. Exosomes derived from human umbilical cord MSCs activate the Sirtuin 1 (*SIRT1*)/Farnesoid X receptor (FXR) pathway in macrophages. Through *SIRT1*-mediated deacetylation of FXR, these exosomes negatively regulate NLRP3 inflammasome assembly and activation, thereby reducing pro-inflammatory cytokine release and alleviating inflammatory bowel disease in mice (124). This mechanism, which centers on the NAD^+^-dependent deacetylase *SIRT1*, directly couples cellular metabolic status with inflammatory signals, representing another mode of exosome-mediated metabolic-inflammatory cross-regulation.

**Figure 2:**
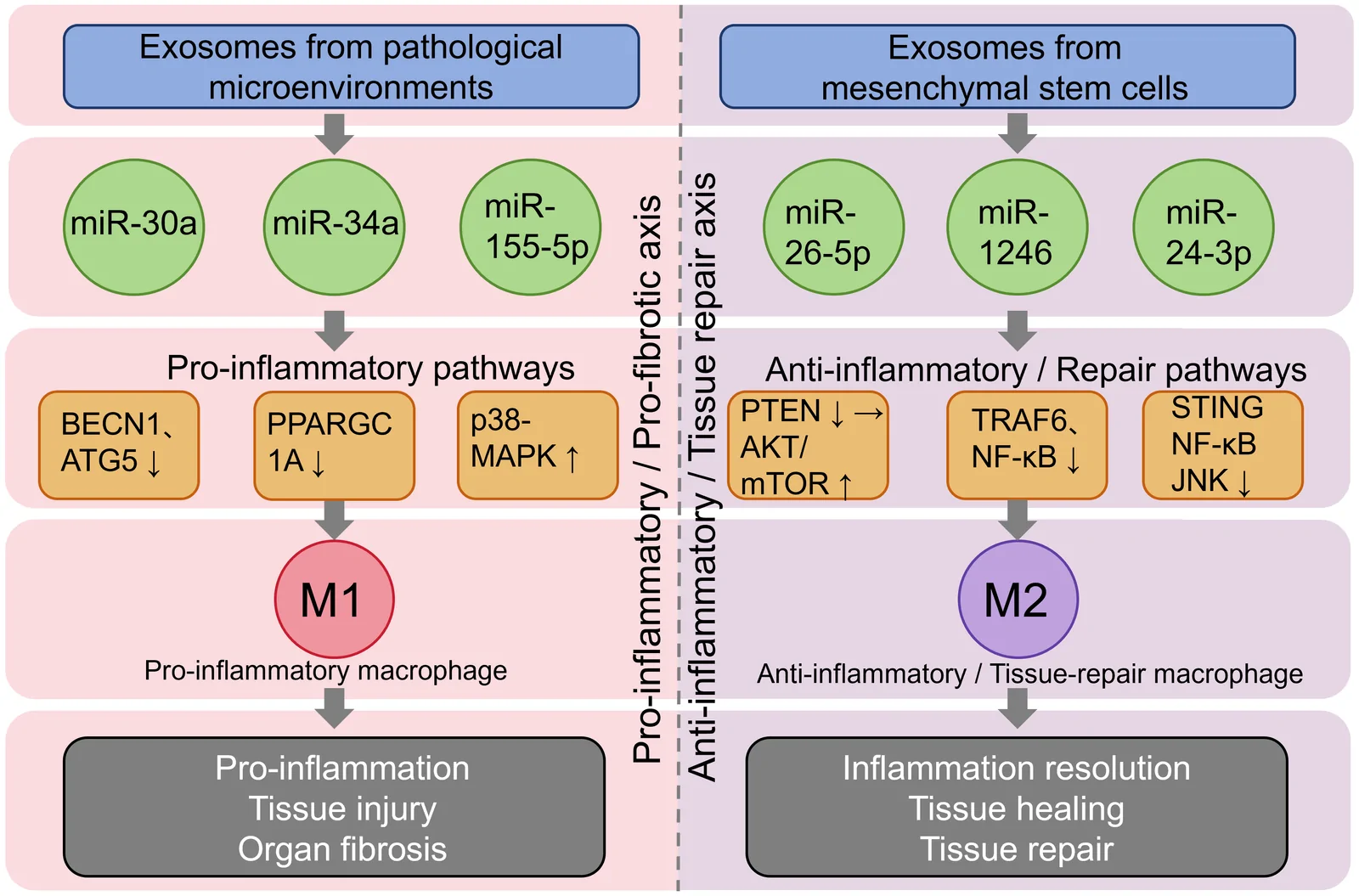
Simplified summary of cargo-specific regulation of macrophage function by exosomes.

In summary, exosome-mediated metabolic reprogramming and classical inflammatory signaling pathways form a complex cross-regulatory network, providing new insights for therapeutic strategies targeting metabolic-inflammatory intersections. However, the aforementioned regulatory effects are not static; the functions of exosomes are highly dependent on their cell type of origin and the surrounding microenvironment. This heterogeneity directly determines the specificity of their regulation of macrophages. To visually illustrate how different exosomal cargos drive macrophage polarization and functional outcomes, we have constructed a simplified summary diagram (**Figure 2**).

This diagram illustrates the complete regulatory axis of “vesicular cargo - targeted signaling pathways - macrophage polarization phenotypes - downstream biological effects.” On the left side, exosomes derived from pathological microenvironments carry miR-30a, miR-34a, and miR-155-5p, which inhibit Beclin 1 (*BECN1*)/Autophagy-related 5 (*ATG5*) and *PPARGC1A*, while activating the p38 MAPK pro-inflammatory pathway. These exosomes induce M1 pro-inflammatory macrophage polarization, ultimately leading to excessive inflammation, tissue damage, and organ fibrosis. On the right side, exosomes derived from mesenchymal stem cells carry miR-26-5p, miR-1246, and miR-24-3p, which regulate the PTEN/AKT/mTOR, *TRAF6*/NF-κB, and Stimulator of interferon genes (STING)/NF-κB/JNK pathways to suppress inflammatory responses. These exosomes drive macrophages toward anti-inflammatory, tissue-repairing M2 polarization, achieving inflammation resolution, tissue healing, and damage repair.

## 5 Heterogeneity and uptake selectivity of exosomes in macrophage regulation

Exosomes are key mediators of intercellular communication and play important roles in regulating macrophage functions. However, exosomes derived from different sources exhibit significant heterogeneity in molecular composition, signal transduction, and functional effects, which directly determines their specific regulation of macrophage polarization and activation. In addition, macrophages, as core members of the mononuclear phagocyte system, exhibit differential internalization efficiency and selectivity toward exosomes, which also influences downstream biological effects. This section systematically compares the heterogeneity characteristics of exosomes from different sources and explores the mechanisms and selectivity of their uptake by macrophages, providing complementary perspectives for understanding exosome-mediated regulation of macrophage functions.

### 5.1 Comparison of heterogeneity among exosomes from different sources

Overall, exosomes derived from different sources display significant heterogeneity in regulating macrophage functions. The direction of their effects, key molecular cargos, and involved signaling pathways are closely associated with the pathological or physiological state of the parent cells (**Table 5**). Based on the microenvironment of origin, these exosomes can be broadly categorized into those derived from the tumor microenvironment, inflammation/injury microenvironment, tissue repair/regeneration microenvironment, metabolic microenvironment, and other sources.

**Table 5:** Comparison of the characteristics of exosomes from different sources in regulating macrophage function.

Exosome source	Core cargo molecules	Signaling pathways	Macrophage regulation	References
Tumor microenvironment				
Hepatocellular carcinoma cells	miR4458HG	miR-100-3p/*ARG1*	Promotes M2 polarization	(113)
	FABP5	PPARγ/PPARα	Induces M2 polarization	(122)
Gastric cancer cells	let-7 g-5p	JAK2/STAT3	Induces M2 polarization	(125)
	BGN	NONO/CXCL10/JAK1/*STAT1*	Induces M2 polarization	(126)
	miR-1911-5p	MYB/AKR1B10/ACC; ARHGEF3	promotes M2 polarization	(127)
Triple-negative breast cancer cells	circ_0076611	STAT6	Induces M2 polarization	(128)
Drug-resistant breast cancer cells	RAB10	IFNAR1/JAK1/*STAT1*	Inhibits M1, promotes M2 polarization	(129)
Breast cancer cells	*MIR4435-2HG*	C/EBPβ	Induces M2 polarization	(130)
Hyperthermia-treated TNBC cells(MDA-MB-231, Hs578T)	HSPB8	MAPK, TNF, IL-17 pathways	Drives M1 polarization, reduces M2 proportion; relieves tumor immune suppression	(131)
Breast cancer cells (ZR-75-1, MCF-7)	PTPRO protein	STAT3/STAT6 dephosphorylation	Induce M1 polarization; reduce M2 TAMs	(132)
Triple-negative breast cancer MDA-MB-231	PEDF protein	Undefined	Reprogram M2 → M1	(133)
Lung adenocarcinoma cells	PD-L1	ER stress pathway (GRP78/CHOP)	Promotes M2 polarization	(134)
Non-small cell lung cancer cells	HMGB1	*TLR4*/NF-κB/IL-6/STAT3	Induces M2 polarization	(135)
Esophageal squamous cell carcinoma cells	NAT10	ac4C modification/*FASN*	Induces M2 polarization, reprograms lipid metabolism	(118)
Melanoma cells	*SND1*, CD47, dsDNA	CD47-SIRPα-cGAS-STING-NF-κB	*SND1*:promote M2; KO:promote M1	(136)
Colorectal cancer cells	HSP90B1	Inhibits M1/activates M2	M1→M2 conversion, inhibits CD8+ T cells	(137)
	LINC01615	RBMX-EZH2 axis	Induces M2 polarization	(138)
Ovarian cancer cells	CMTM4	NF-κB/ICAM1	Induces M2 polarization	(139)
LNCaP prostate cancer cells, PCa patient urine exosomes	miR-203	Pro-inflammatory cytokine axis	Induces M1 polarization, suppresses M2	(140)
HNSCC (HPV+/HPV−)	AP1/NF-κB related transcripts	*STAT1*, NF-κB, AP-1 signaling axis	HPV−:drive M1; HPV+: induce mixed M1/M2	(141)
Pancreatic adenocarcinoma PANC-1 cells	miR-34a microRNA	*SOCS3*/JAK/*STAT1*	Inhibit M2 polarization;	(142)
Inflammatory/injury microenvironment				
Serum from septic mice		PD-1↑/*KLF4*	Inhibits M2 polarization	(143)
Silica-stimulated macrophages	HMGB3	STAT3/MAPK/NF-κB/CCR2	Promotes M1 polarization and recruitment	(30)
Inflammatory fibroblast-like synoviocytes		*HIF1A*-glycolysis axis	Promotes M1 polarization	(114)
Netrophils from Behçet’s disease patients	miR-122-5p (deficient)	miR-122-5p/IRF5/IFN-β/STAT1	Healthy: inhibit M1; BD: lack this function	(144)
K. pneumoniae-infected epithelial cells	Phosphatidylcholine	NF-κB	Induces M1 polarization	(145)
Tissue repair/regeneration microenvironment				
Human umbilical cord MSCs	miR-21-5p	*SPRY2*/ERK	Promotes M2 polarization	(19)
UC-MSCs (ALKBH5-overexpressing)	ALKBH5	m6A/*TRAF6*/NF-κB/STAT1	Promotes M2 polarization	(20)
iPSC-derived neural stem cells	let-7b-5p	LRIG3/NLRP3/Caspase-1/GSDMD	Inhibits pyroptosis	(37)
Bone marrow MSCs	lncRNA SNHG12	GPX4, inhibits ferroptosis	Inhibits ferroptosis	(146)
Regulatory T cells	IL-10, TGF-β, miR-146a	*TLR4*/MAPK/NF-κB, PI3K-Akt	Inhibits M1, promotes M2	(147)
Dental follicle stem cells	miR-140-3p	DNMT1/SOCS1/NF-κB	Indirectly inhibits M1, promotes M2	(148)
Metabolic microenvironment				
Renal tissue from diabetic nephropathy mice	miRNA-34a	*PPARGC1A*	Promotes M1 polarization	(13)
HG+oxLDL-treated endothelial cells	NEDD4L	Ubiquitinates IκBα/PPARγ, activates NF-κB	Promotes M1 polarization	(149)
Calcium oxalate-treated tubular cells	miR-93-3p	NFAT5/Akt1/NIK/NF-κB2	Promotes M1 polarization	(150)
Palmitic acid-stimulated macrophages	miR-3064-5p	Targets IκBα, activates NF-κB	Promotes M1 polarization	(151)
Other sources				
Stem cells from human exfoliated deciduous teeth	TGF-β, IL-10, CAT, SOD	*TLR4*/NF-κB, STAT3/6, Nrf2	Promotes M2 polarization	(152)
Human breast milk	lncRNA FP671120.4	ELAVL1/Nrf2/HO-1	Inhibits M1 polarization	(153)

In the tumor microenvironment, the vast majority of exosomes induce macrophages toward M2 polarization. This pattern has been summarized in a systematic review by Kalluri and LeBleu, with the core mechanism involving multidimensional regulation of macrophage polarization by exosomal non-coding RNAs, metabolic enzymes, and immunomodulatory proteins (154) (new reference added). Nevertheless, a minority of exosomes can drive M1-like polarization switching and inhibit tumor proliferation. For example, exosomes derived from ZR-75-1 and MCF-7 cells carry Protein tyrosine phosphatase receptor type O (PTPRO), which induces M1-like polarization of THP-1-derived macrophages and inactivates the STAT signaling pathway by inhibiting STAT3/STAT6 phosphorylation in macrophages, thereby blocking breast cancer cell invasion and migration (132). Exosomes with high Pigment epithelium-derived factor (PEDF) expression secreted by human breast cancer MDA-MB-231 cells can reprogram primary human M2 macrophages toward the M1 phenotype (133). Exosomes secreted by LNCaP prostate cancer cells, which are highly loaded with miR-203, induce M0 macrophage polarization toward the M1 phenotype; *in vivo* studies using nude mouse xenograft models have confirmed that these exosomes significantly inhibit prostate cancer tumor growth (140). Exosomes derived from B16F10 melanoma cells with Staphylococcal nuclease and tudor domain containing 1 (*SND1*) deficiency are more readily internalized by macrophages due to the scarcity of CD47, and drive M1 macrophage polarization through the double-stranded DNA (dsDNA)-cGAS-STING pathway, thereby inhibiting melanoma lung metastasis (136). Exosomes from OCT1-expressing human HPV-negative HNSCC cells induce M1 polarization of THP-1 macrophages, whereas exosomes from HPV-positive 93VU147T and UDSCC2 cells induce a mixed M1/M2 phenotype (141).

Furthermore, the same miRNA may exert opposite functions in different tumor types. For instance, exosomal miR-34a derived from pancreatic adenocarcinoma PANC-1 cells targets *SOCS3* to inhibit M2 macrophage polarization and block pancreatic cancer malignant progression. In contrast, exosomal miR-34a derived from renal tubular epithelial cells of db/db mice with diabetic nephropathy targets *PPARGC1A* to induce M1 macrophage polarization and aggravate renal tubulointerstitial fibrosis (13, 142). This functional divergence may arise from cell type-dependent target gene profiles, differences in exosomal cargo composition, and synergistic regulation by microenvironmental signals.

In the inflammation and tissue injury microenvironment, exosomal regulation of macrophages exhibits bidirectional effects, either promoting M1-like pro-inflammatory polarization or suppressing it, depending on the disease type, disease stage, and cell origin. For example, exosomes from different sources drive M1 polarization and inflammatory responses by carrying molecules such as HMGB3 (30), miR-221-3p (49), lncRNA MIR17HG (55), miR-155-5p (70), and lncRNA H19 (103). Conversely, exosomes from LPS-pretreated rat primary bone marrow-derived MSCs, which are enriched with miR-222-3p, promote macrophage switching toward the M2 phenotype and suppress M1 polarization, thereby alleviating inflammatory injury in transplanted tissues (53). Exosomal circ-CBLB derived from primary rheumatoid arthritis fibroblast-like synoviocytes inhibits M1 polarization through the TLR3/TRAF3 axis (100). Exosomes secreted by primary peripheral blood neutrophils from patients with Behçet’s disease exhibit downregulated expression of the core anti-inflammatory molecule miR-122-5p, which relieves the suppression of human primary macrophages through the IRF5/IFN-β autocrine pathway, ultimately mediating excessive macrophage activation (144).

In the tissue repair and regeneration microenvironment, exosomes generally exhibit functional characteristics of promoting M2 polarization and suppressing inflammation, with mesenchymal stem cell-derived exosomes being the most typical. Engineered or preconditioned exosomes from different sources carry molecules such as miR-24-3p (97), miR-21-5p (19), miR-26-5p (18), miR-135b (60), let-7b-5p (37), lncRNA SNHG12 (146), and circRNA ATG7 (99). These exosomes regulate signaling pathways including *SIRT1*/FXR, PTEN/AKT, STING/NF-κB, GPX4, and NLRP3, thereby suppressing M1 polarization, promoting M2 polarization, and inhibiting macrophage pyroptosis and ferroptosis to exert tissue-repairing functions.

In the metabolic microenvironment, exosomes are primarily involved in inflammation regulation associated with metabolic disorders. Under pathological conditions such as obesity, diabetes, and hyperlipidemia, exosomes from different sources carry molecules including the miR-200 family (155), miR-1246 (96), NEDD4L (149), miR-93-3p (150), and miR-3064-5p (151), as well as miRNA-34a (13). These exosomes activate pathways such as NF-κB and PI3K/AKT/mTOR, inducing M1 polarization and pro-inflammatory phenotypes, and participating in the development of insulin resistance, diabetic nephropathy, nephrolithiasis, and metabolic inflammation. Notably, exosomes derived from adipose stem cells of lean individuals can promote M2 polarization through the FTO/RUNX1T1/*TRAF6*/NF-κB axis, suggesting that the metabolic status of donor cells influences exosomal function (96). Exosomes from other sources, such as those derived from stem cells from human exfoliated deciduous teeth (SHED) (152) and breast milk (153), can also suppress M1 polarization and promote macrophage shifting toward the M2 anti-inflammatory phenotype through unique molecular mechanisms, further demonstrating the breadth of exosomal source heterogeneity.

In summary, exosomes derived from different sources exhibit significant differences in regulating macrophage polarization direction, effector molecules, and signaling pathways. This heterogeneity depends not only on the type and status of the parent cells but also on the microenvironment in which the macrophages reside. However, to gain a deeper understanding of the underlying principles governing this process, further investigation into the specific mechanisms of macrophage uptake of exosomes and their selectivity is warranted.

### 5.2 Mechanisms and selectivity of macrophage uptake of exosomes

The process by which exosomes are taken up by macrophages involves complex signal recognition, internalization pathways, and multiple influencing factors, and exhibits significant heterogeneity and selectivity. The uptake of exosomes by macrophages is not a random process but a highly controlled event of recognition and internalization. Mathieu et al. systematically summarized the molecular mechanisms by which exosomes are taken up by target cells, noting that this process depends on specific recognition between exosomal surface ligands and target cell surface receptors, and is jointly influenced by donor cell status and microenvironmental factors (156).

#### 5.2.1 Signaling and uptake modes of exosomes

The interaction between macrophages and exosomes involves two levels: uptake, i.e., the physical internalization of exosomes by macrophages; and signaling, i.e., the functional responses triggered after the molecular information carried by exosomes enters or is presented to macrophages. Enhanced uptake efficiency does not necessarily equate to enhanced functional reprogramming; the ultimate effect depends on the delivered cargo molecules and the signaling pathways they activate. As shown in **Figure 2**, the interaction between macrophages and exosomes can be summarized into the above two steps. At the uptake level, exosomes enter cells through receptor-mediated endocytosis, phagocytosis, membrane fusion, and other pathways. At the signaling initiation level, membrane molecules on the exosomal surface can directly bind to macrophage surface receptors, triggering uptake-independent signal transduction as well as uptake-dependent intracellular signaling activation.

In terms of uptake modes, macrophages primarily internalize exosomes through endocytosis (including receptor-mediated endocytosis and phagocytosis), while membrane fusion serves as a supplementary pathway that directly releases exosomal contents into the cytoplasm. Receptor-mediated endocytosis is the major route for exosome entry into macrophages, and this process depends on the recognition of exosomes by macrophage surface receptors. *TLR4* is a key receptor for recognizing exosomal HMGB1 (135). TLR3 recognizes exosomal circ-CBLB in rheumatoid arthritis and participates in signal activation (100). CD169^+^ macrophages can mediate specific uptake of B cell-derived exosomes by recognizing sialic acid on the exosomal surface (157), suggesting a unique role for specific macrophage subsets in the uptake of immune cell-derived exosomes.

**Figure 3:**
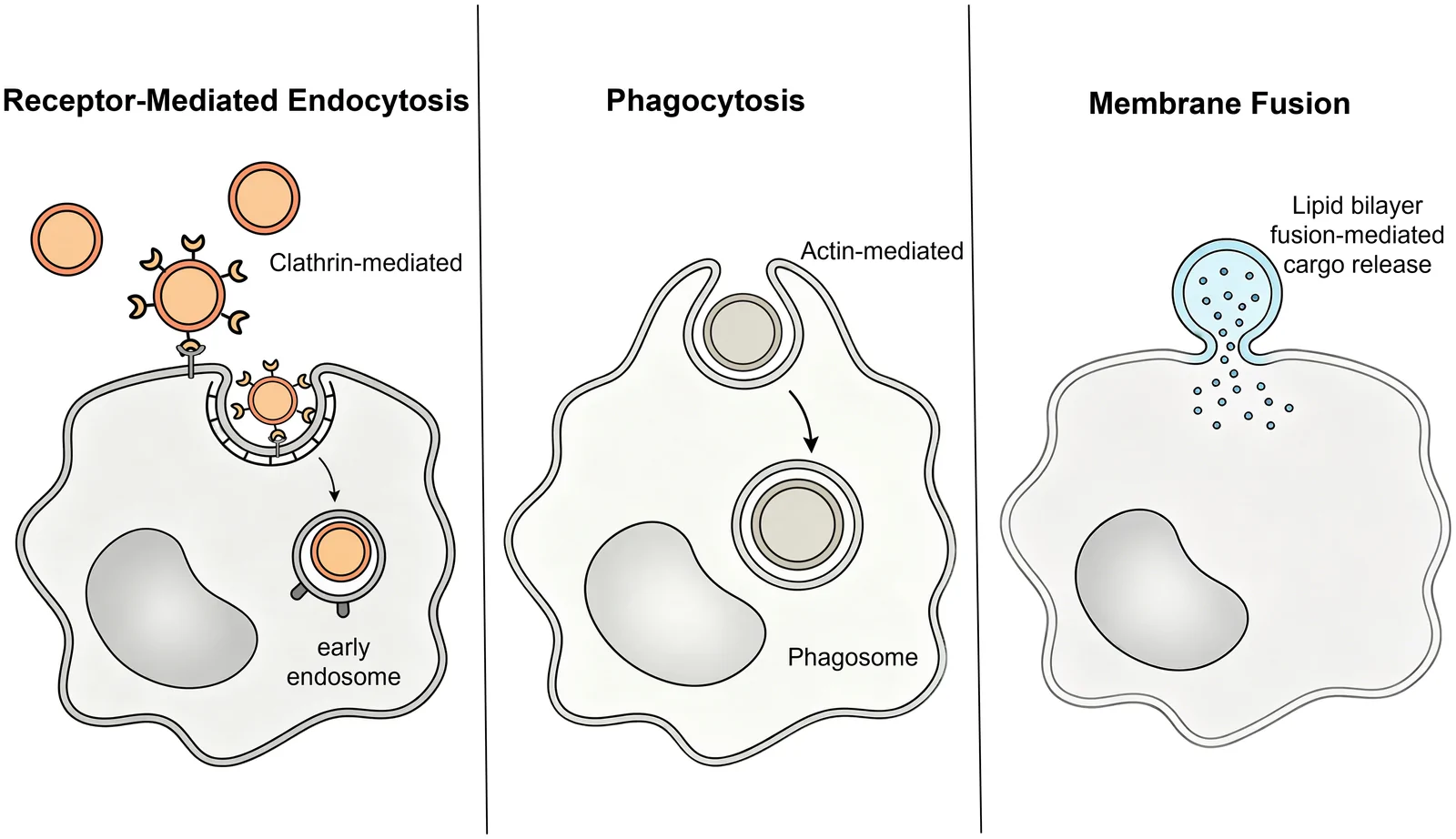
Mechanisms of exosome uptake by macrophages.

Phagocytosis, as another important form of endocytosis, has also been clearly demonstrated to participate in macrophage uptake of exosomes in multiple studies. Examples include exosomes derived from patients with endometriosis (64), renal tissue exosomes in diabetic nephropathy (13), exosomes derived from mouse RAW264.7 cells (158), lemon-derived exosome-like nanoparticles (159), and blood-derived exosomes from septic mice (143), among others. Membrane fusion, as a supplementary pathway for uptake and cargo delivery, can directly release exosomal miRNAs, proteins, and other contents into the cytoplasm. For example, engineered exosomes modified with platelet membranes (160) and exosomes secreted by AsPC-1 pancreatic cancer cells (161) have been shown to achieve cargo delivery through this pathway. These three pathways represent different modes of the uptake step, and their common outcome is to enable exosome entry into or contact with macrophages. However, the uptake process itself does not directly determine the ultimate functional outcome. The three major pathways of macrophage uptake of exosomes are illustrated in **Figure 3**.

This figure illustrates three classical molecular mechanisms by which macrophages capture and internalize exosomes. Left: receptor-mediated endocytosis. Ligands on the exosomal membrane surface specifically bind to receptors on the macrophage membrane, followed by clathrin-mediated membrane invagination and internalization, ultimately forming early endosomes. Middle: phagocytosis. Macrophages remodel the cell membrane through actin cytoskeleton rearrangement, engulfing exosomes to form phagosomes for internalization. Right: membrane fusion pathway. The lipid bilayer of exosomes directly fuses with the macrophage plasma membrane, releasing internal bioactive cargo directly into the cytoplasm without passing through the endosomal vesicle stage.

**Figure 4:**
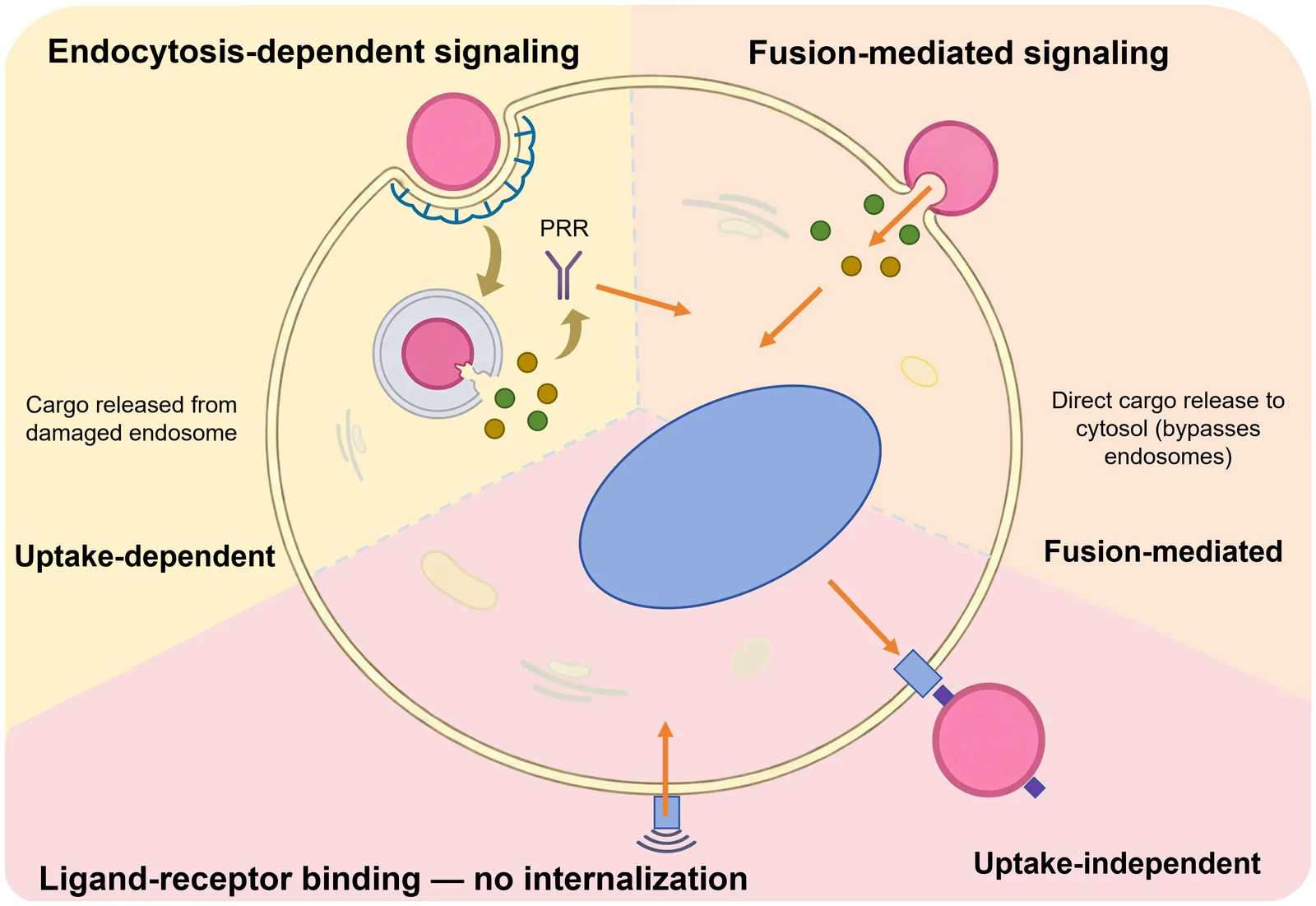
Schematic diagram of the main pathways for exosome-macrophage signaling.

At the signaling level, exosomes transmit signals to macrophages through three modes, of which the first two are uptake-dependent and the third is uptake-independent: (1) uptake-dependent intracellular signaling activation — after exosomes are internalized by macrophages via endocytosis, their carried nucleic acids or proteins are recognized by intracellular pattern recognition receptors (PRRs), thereby initiating downstream signaling pathways related to inflammation, immunomodulation, or polarization (162); (2) membrane fusion-dependent intracellular signaling activation — after the exosomal membrane directly fuses with the macrophage plasma membrane, miRNAs, proteins, and other contents are released directly into the cytoplasm, bypassing the endosomal escape bottleneck and directly participating in intracellular signaling regulation (163); (3) uptake-independent direct surface receptor modulation — membrane molecules on the exosomal surface can directly bind to corresponding receptors on the macrophage plasma membrane without entering the cell, initiating intracellular signal transduction. For example, CD47 on the surface of exosomes derived from visceral white adipose tissue of obese mice can bind to Signal regulatory protein alpha (SIRPα) on the macrophage surface, thereby modulating phagocytic efficiency (155). Among these three modes of signal transduction, the first two rely on exosomal delivery of functional cargo into the cell, whereas the third independently initiates signal transduction without requiring uptake, representing a rapid signaling event directly mediated by exosomal surface molecules, as illustrated in **Figure 4**.

This figure comprehensively illustrates three signaling pathways by which exosomes regulate macrophages. Upper left (orange area): endocytosis-dependent signaling pathway. After exosomes are internalized via endocytosis, damaged endosomes release vesicular contents, and intracellular PRRs recognize the cargo and activate downstream signals, mediating macrophage phenotypic remodeling. Upper right (light yellow area): membrane fusion-dependent signaling pathway. The exosomal lipid membrane directly fuses with the macrophage plasma membrane, bypassing the endosomal stage and releasing bioactive cargo directly into the cytoplasm to activate cascade reactions. Bottom (purple area): uptake-independent ligand-receptor signaling pathway. Exosomal surface ligands bind only to receptors on the macrophage membrane, rapidly initiating membrane-associated signal transduction without requiring vesicular internalization. The orange area represents uptake-dependent signaling, while the purple area represents signaling that does not require vesicular internalization. The core difference among these three modes is that cargo-mediated functional effects require physical internalization of exosomes into the cell, whereas signals triggered solely by receptor binding do not require internalization. Therefore, enhanced exosome uptake does not necessarily equate to enhanced macrophage functional remodeling.

Uptake merely represents physical contact and internalization of exosomes by macrophages, whereas the direction and intensity of signal transduction are jointly determined by the type, concentration, and signaling nodes targeted by the cargo molecules. These two processes together constitute the complex signaling interaction network between exosomes and macrophages.

#### 5.2.2 Factors affecting uptake

The efficiency of exosome uptake by macrophages is regulated by multiple factors, including exosomal surface molecules, macrophage subtypes, the microenvironment, and cell preconditioning.

The exosomal surface molecular profile is one of the key factors affecting uptake. CD47 is the most representative signaling molecule, and its high expression on the exosomal surface can significantly inhibit macrophage phagocytosis (97, 136). Conversely, some surface molecules can promote uptake. For example, CD44 is involved in the recognition and binding between exosomes and macrophages (164). High expression of CD105 on urinary exosomes from patients with lupus nephritis promotes macrophage uptake and M1 polarization (165). Based on the expression profiles of multiple receptors on the macrophage surface, researchers have developed corresponding engineered targeting strategies. M1 macrophages constitutively express high levels of the mannose receptor CD206. Mannose-modified mesenchymal stem cell exosomes can be specifically internalized through CD206-mediated endocytosis, significantly enhancing macrophage uptake levels, and this targeting effect can be abolished by free mannose competitive inhibition (166). In the tumor microenvironment, M2-type tumor-associated macrophages specifically express high levels of folate receptor beta (FRβ). This receptor mediates preferential internalization of folate-modified carriers by M2 macrophages. Whether using artificial lipid nanoparticles or ginger-derived exosome-like nanoparticles, folate modification significantly enhances targeted uptake by M2 macrophages and reverses the pro-tumor phenotype of macrophages through regulation of the IRF3 or PI3K-AKT pathways (167, 168). Engineered exosomes carrying anti-CD16 antibodies can achieve selective endocytic delivery to CD16^+^ macrophages (169).

Whether M1 and M2 macrophages exhibit differential uptake efficiency for exosomes currently lacks quantitative consensus. A few existing studies provide preliminary indirect clues. Choo et al. observed that M2 macrophages exhibit higher uptake efficiency for M1-derived exosome-like nanovesicles compared to tumor cells (170). Kim et al. found that internalization of M2-derived exosomes by M1 macrophages occurs in a time- and dose-dependent manner (171). Zhu et al., from a functional response perspective, noted that M2 macrophages demonstrate higher functional integration capacity for exosomal signals through the CSF-1/CSF-1R axis (172). It is worth noting that the above studies focus on functional responses rather than uptake efficiency per se, and each employs different experimental systems and quantitative metrics, making direct comparisons difficult. Furthermore, it is necessary to clearly distinguish between “uptake efficiency” and “functional response”, as the former is not equivalent to the latter.

The regulatory role of microenvironmental factors in macrophage uptake of exosomes should also not be overlooked. Pathological conditions such as inflammation, hypoxia, and hyperglycemia have all been reported to enhance macrophage uptake of exosomes. In the inflammatory microenvironment, exosomes from the circulation of septic mice can activate the NF-κB pathway in macrophages, whereas exosomes from normal mice lack this effect (55, 143). In a muscle injury model, exosomes derived from fibro-adipogenic progenitors at different time points after injury exhibited significantly higher uptake efficiency and pro-regenerative function compared to those from uninjured or early-injury stages (173). Exosomes from various pathological conditions, including diabetic nephropathy (13, 99), lupus nephritis (165), Crohn’s disease (58), and osteoarthritis (60), all exhibit different post-uptake functions compared to exosomes from normal states. The hyperglycemic microenvironment also affects exosome uptake. For example, urinary stem cell-derived exosomes are preferentially taken up in the kidneys of diabetic nephropathy mice (99). Under hyperglycemic pathological conditions, the anti-inflammatory response of macrophages to exosomes is significantly reduced, though melatonin-modified stem cell exosomes can restore the macrophage repair phenotype switching capacity (65).

Hypoxia is a common feature of the tumor microenvironment and the injury microenvironment, and can significantly alter exosome secretion and cargo composition, thereby affecting macrophage uptake efficiency and functional responses. Studies have shown that hypoxia enhances exosome release from human primary glioblastoma cells and upregulates the expression of miR-25/93 in exosomes. These exosomes can be efficiently taken up by macrophages, subsequently inhibiting cGAS-STING pathway activity and inducing immunosuppression (174). Under intermittent hypoxia, exosomal miR-106a-5p derived from non-small cell lung cancer A549 cells promotes M2 macrophage polarization by downregulating PTEN and activating the STAT3 pathway (175). Exosomes derived from hypoxic preconditioned H9c2 cardiomyocytes can be phagocytosed by RAW264.7 macrophages and induce M2 polarization (176). Exosomes derived from hypoxic preconditioned rat primary mesenchymal stem cells are efficiently taken up by alveolar macrophages and regulate macrophage polarization and oxidative stress through delivery of Solute carrier family 25 member 3 (SLC25A3) (177). These findings indicate that hypoxia not only alters the molecular composition of exosomes but also amplifies their biological effects by enhancing macrophage uptake capacity.

Cell preconditioning can also significantly affect exosome uptake and function. For example, LPS preconditioning increases the secretion and protein content of mesenchymal stem cell-derived exosomes (39), and the uptake efficiency by macrophages is significantly higher than that of non-preconditioned exosomes (53). After emodin preconditioning, the inflammatory response induced by pancreatic exosomes secreted by primary injured rat pancreatic acinar cells is significantly reduced (25). Hyperthermia at 43 °C not only increases exosome release from triple-negative breast cancer cells but also enhances macrophage uptake of exosomes, relying on enriched Heat shock protein family B (small) member 8 (HSPB8) to drive M1 reprogramming of macrophages (131). These preconditioning strategies alter the surface characteristics and cargo composition of exosomes, enhancing their recognition and internalization by macrophages, and providing new ideas for optimizing exosome-based therapies.

#### 5.2.3 Source-dependence of uptake efficiency and functional differences

The source of exosomes determines their uptake efficiency by macrophages and their subsequent functions. Exosomes derived from tumor cells are more readily taken up by macrophages than those from normal cells, and they induce polarization toward the pro-tumor M2 phenotype. For example, exosomes from hepatocellular carcinoma cells (51, 68, 87, 102), gastric cancer cells (69, 78, 125, 126), lung cancer cells (12, 63, 135), esophageal squamous cell carcinoma cells (72, 118), breast cancer cells (73, 88, 130), colorectal cancer cells (47, 138), ovarian cancer cells (139, 178), renal cancer cells (90, 179), glioma stem cells (45), and cholangiocarcinoma cells (180) all exhibit significantly higher uptake efficiency compared to their corresponding normal cell-derived exosomes.

Engineered modification can significantly alter the uptake efficiency and function of exosomes. For example, engineered exosomes modified with folate (167, 168), mannose (166), platelet membrane coating (160), or antibodies (158, 169) exhibit much higher targeted uptake efficiency by macrophages than unmodified exosomes. Similarly, engineered exosomes with altered cargo content, such as those overexpressing specific miRNAs or genes, exhibit stronger functional effects after uptake compared to unmodified exosomes (47, 60, 67, 74, 129, 181, 182). Conversely, knockdown or knockout of specific functional molecules in exosomes—such as *SND1 (*136),*THBS1* (178), *MEG3* (86), circ_0001715 (93), LCN2 (16), miR-221-3p (49),*SLC16A1-AS1* (87), circ_0076611 (128),*NEAT1* (45), Rmrp (105)—significantly attenuates their function, even though their uptake efficiency may remain unchanged.

When uptake efficiency is comparable, function is determined by cargo composition. For example, under equivalent uptake conditions, exosomes from the rat pancreatic acinar cell line AR42J, which are enriched with miR-125b-5p, exhibit stronger inhibitory effects on M2 polarization (101). Exosomes from human primary cancer-associated fibroblasts (CAFs) enriched with miR-320a induce significantly stronger M2 polarization effects (74). Exosomes derived from mouse CT-26 colon cancer cells, which are rich in Heat shock protein 90 beta family member 1 (HSP90B1), drive more pronounced M2 polarization (137). The function of exosomes is essentially determined by the miRNAs, proteins, and lncRNAs they carry; uptake merely provides the channel for these cargo molecules to enter macrophages.

In summary, macrophage uptake of exosomes is a highly controlled and heterogeneous process. Exosomal surface molecules, macrophage receptor status, the microenvironment, and the state of the parent cells collectively determine the efficiency and pathways of uptake. The uptake efficiency, together with the characteristics of the cargo, jointly determines the direction and intensity of exosomal regulation of macrophage functions. Understanding these mechanisms has important guiding significance for the development of novel exosome-based therapeutic strategies. Although substantial progress has been made in basic research, exosome-based therapies still face numerous challenges before clinical application.

### 5.3 Challenges in EV nomenclature, methodology, and cross-study integration

According to the MISEV2023 guidelines (162), the terms “exosomes” and “microvesicles” should only be used in contexts where their biogenesis can be clearly demonstrated. However, the vast majority of primary studies cited in this review rely on particle size or isolation method differences without verifying endosomal origin, and thus cannot distinguish between different EV subtypes, including microvesicles, apoptotic bodies, and other sEVs. Consequently, the currently described mechanistic effects attributed to exosomes may actually reflect the combined outcomes of multiple subtypes, whose individual contributions cannot be differentiated.

Importantly, different exosome isolation methods—including ultracentrifugation, size-exclusion chromatography, and precipitation-based kits—each recover EV subpopulations that differ significantly in purity, yield, and functional integrity (183, 184). This methodological heterogeneity makes direct cross-study comparisons extremely challenging. Future studies should adhere to the MISEV2023 framework (4): (i) report isolation parameters in detail, (ii) characterize EV preparations using multiple complementary methods (185), (iii) acknowledge the limitations of subtype classification, and (iv) whenever possible, validate key findings using at least two independent isolation methods. Current exosome research remains predominantly descriptive and observational; methodological rigor of this kind is a fundamental prerequisite for building a coherent and clinically translatable body of knowledge.

## 6 Progress in clinical translation

Exosomes, as natural nanoscale vesicular carriers, possess advantages including low immunogenicity, high biocompatibility, and engineering modifiability, demonstrating broad application prospects in regulating macrophage functions and treating immune-related diseases. However, the translation from basic research to clinical application still faces multiple core bottlenecks. Systematically elucidating these issues and exploring targeted solutions is critical for advancing the clinical translation of exosome-based therapies.

### 6.1 Challenges and corresponding strategies in clinical translation

At the level of applied translation, exosomes as potential therapeutic carriers for immune- and cancer-related diseases (186) face multiple engineering and regulatory bottlenecks that urgently require systematic resolution.

#### 6.1.1 Standardized production and quality control systems

The lack of standardized production and quality control systems is the primary bottleneck in the clinical translation of exosomes. Currently, there is no unified standard for exosome isolation and purification methods, resulting in significant batch-to-batch variations in purity, yield, and functional activity (185). The diversity of manufacturing technologies makes standardization challenging and leads to fragmented regulatory frameworks (187). Furthermore, there is no industry consensus on issues such as long-term storage stability of exosomes and activity evaluation indices. The International Society for Extracellular Vesicles released the MISEV2023 guidelines, providing researchers with an updated reference framework for exosome production, isolation, and characterization, ranging from basic to advanced methods (4). It should be emphasized that MISEV2023 is positioned as a reporting standard aimed at improving transparency and methodological rigor in EV research; it does not address product quality control or GMP standards. There remains a considerable gap between MISEV2023 and the establishment of operational GMP-grade clinical production standards.

To address this bottleneck, researchers are actively developing large-scale exosome production processes that comply with GMP standards. A recent review systematically discussed the obstacles encountered when translating MSCs and their exosomes from research platforms into GMP-compliant advanced therapy medicinal products, and proposed solutions including the use of automated closed systems for real-time quality control monitoring to facilitate translation (188). Another study provided a detailed description of GMP-based upstream and downstream large-scale EV production processes, encompassing cell culture, purification, and surface bioengineering and post-processing technologies (183). This study emphasized that the complex interplay among strict GMP guidelines, raw materials, bioreactor production, and isolation practices is essential for standardized and consistent GMP production (184). In addition, establishing multidimensional quality control standards covering exosome origin, purity, particle size, surface markers, and functional activity, as well as exploring lyophilization preservation and long-term storage stability evaluation methods, are important directions for ensuring batch-to-batch stability and reproducibility (183). The above studies provide feasible technical pathways for GMP production of exosomes from the perspectives of production process standardization and quality control system establishment, although considerable distance remains from large-scale clinical application.

#### 6.1.2 Targeted delivery efficiency

Insufficient targeted delivery efficiency severely limits the therapeutic efficacy of exosomes. After intravenous administration, native unmodified exosomes entering the circulation are rapidly captured and cleared by the mononuclear phagocyte system in the liver and spleen, making it difficult for them to effectively infiltrate and act on macrophages at the lesion site. Their enrichment efficiency in target tissues is low, and they’re *in vivo* clearance rate is relatively high (189). The mechanisms of vesicular clearance mediated by macrophage polarization and plasma protein corona remodeling have been systematically elucidated (190). *In vivo* quantitative data from radionuclide PET imaging have directly confirmed the preferential accumulation of exosomes in the liver and spleen, with low enrichment in peripheral target tissues (191).

Engineered exosome modification is a core strategy for enhancing targeting. In terms of surface modification, the introduction of targeting ligands can enhance the delivery efficiency of exosomes to specific macrophage subsets. For example, mannose-modified exosomes utilize the high expression of CD206 receptors on alveolar macrophages to achieve specific targeted uptake (166). In terms of loading functional molecules, adipose stem cell-derived exosomes loaded with miR-1246 promote macrophage polarization toward the M2 phenotype and alleviate obesity-associated metabolic disorders by targeting and inhibiting *TRAF6* expression and blocking NF-κB signaling pathway activation (96). Adipose stem cell-derived exosomes loaded with icariin significantly inhibit M1 macrophage polarization and reduce inflammatory responses by suppressing the *TLR4*/*MyD88*/NF-κB signaling pathway (51). B cell-derived exosomes have been successfully used to deliver miRNA-155 inhibitors to macrophages (192), demonstrating the potential of immune cell-derived exosomes as targeted drug delivery vehicles.

In terms of composite applications of materials and exosomes, Jiang et al. (40) constructed FM-Exo nanocomposite hydrogels by combining MXene two-dimensional nanomaterials with M2 macrophage-derived exosomes. This system possesses temperature sensitivity, injectability, self-healing properties, and sustained exosome release capability. In a high-glucose microenvironment, FM-Exo induces M2 macrophage polarization through activation of the PI3K/AKT pathway, promotes fibroblast proliferation and migration, and enhances endothelial cell angiogenesis. In a mouse model of full-thickness diabetic skin defects, this composite effectively regulated macrophage polarization status, alleviated inflammation, and promoted vascularization and collagen deposition. The core innovation of this study lies in the material design; however, the specific miRNAs or proteins in M2-Exo that mediate the polarization effect have not been identified, and the long-term *in vivo* safety of MXene materials requires systematic evaluation.

In terms of constructing composite delivery systems, biomimetic exosome-silica nanohybrids achieve targeted enrichment in inflamed joints in a rheumatoid arthritis model, significantly alleviating joint swelling and bone damage (193). Hydrogel-loaded exosomes enable sustained release delivery and accelerate wound repair in diabetic wound healing through continuous regulation of macrophage polarization (159). In terms of administration route optimization, aerosolized inhalation of pulmonary microvascular endothelial cell-derived exosomes can effectively target pulmonary macrophages, significantly improving exosome enrichment efficiency in lung tissue (194). The above strategies, ranging from exosome self-modification and composite system construction to administration route optimization, constitute a multi-level solution for targeted delivery.

#### 6.1.3 Immunogenicity and safety

Allogeneic exosomes may carry donor-derived antigens or genetic material, potentially triggering immune rejection reactions. Engineered exosomes with surface-modified molecules or loaded exogenous drugs may also exhibit immunogenicity. Currently, safety data on exosomes, including long-term toxicity, tumorigenic risk, and biodistribution, remain insufficient, and their immunogenicity and safety profiles require systematic evaluation (195).

To reduce immunogenicity risks, researchers are actively exploring alternative sources with a priority on safety. Plant-derived exosome-like nanoparticles—such as those from ginseng, platycodon, and salvia miltiorrhiza—offer advantages due to their natural origin and favorable biocompatibility. For example, fig-derived exosome-like nanoparticles have demonstrated good biosafety in animal experiments (196), and Achyranthes bidentata-derived nanovesicles show no obvious organ toxicity (197). These natural source-derived exosomes may serve as promising candidates for further development with safety as a priority (198–200). However, the long-term safety of these naturally derived exosomes still requires validation through standardized toxicological studies.

Therefore, establishing a systematic preclinical safety evaluation framework is essential. First, pharmacokinetic and biodistribution studies of exosomes should be conducted to clarify their metabolic pathways and accumulation organs *in vivo* (201). Second, long-term toxicity studies should be performed to assess the potential tumorigenic risk, immunogenicity, and organ toxicity of exosomes (202). Most importantly, safety evaluation standards suitable for different administration routes need to be established, particularly for local administration routes such as aerosolized inhalation, intra-articular injection, and intraspinal injection, which have special safety requirements (203).

#### 6.1.4 Scalable production and cost

Scalable production and cost control represent realistic obstacles to clinical application. Exosomes have low yields and high production costs, making it difficult to meet clinical demands. The lack of large-scale preparation methods and adequate quality control approaches severely limits the development of therapeutic products and their global clinical application (183, 188).

To overcome this obstacle, efficient and economical production processes should be developed. On one hand, optimization strategies for cell culture conditions—such as three-dimensional culture, hypoxic preconditioning, and genetic engineering—should be explored to increase exosome yield and functional activity (188). On the other hand, high-throughput isolation and purification technologies, such as tangential flow filtration and the combination of size-exclusion chromatography with affinity chromatography, should be developed to improve purification efficiency and product purity (183). In addition, cost-effectiveness analysis models for exosome-based drugs should be established to provide a basis for clinical pricing and reimbursement decisions. The concept of “point-of-care preparation” proposed by Zou et al. allows flexible adjustment of the ratio of exosomes to drugs and administration routes according to patient conditions, providing a feasible approach for personalized therapy and cost control (204).

#### 6.1.5 Common limitations in current clinical translation

Beyond the specific challenges at each stage described above, exosome clinical translation research also faces several common limitations that persist throughout the process. At the methodological level, there is a lack of standardized protocols for exosome isolation, purification, characterization, and nomenclature reporting. Products obtained through ultracentrifugation, size-exclusion chromatography, and polymer precipitation differ significantly in purity and integrity, resulting in poor reproducibility across different laboratories. Most studies broadly refer to sEV-enriched products as “exosomes” without rigorously verifying their endosomal origin, and the interchangeable use of terms such as “exosomes,” “microvesicles,” and “sEVs” further exacerbates inter-study heterogeneity. At the evidence level, the vast majority of conclusions are derived from cell lines or animal models, with relatively few validations using primary human cells, and only a handful have entered clinical trials. Even among those that have entered clinical studies, sample sizes are generally small and randomized controls are lacking, resulting in limited levels of evidence. At the translational level, existing studies heavily rely on rodent models such as mice, which differ significantly from the human immune microenvironment and pathophysiology; therapeutic effects observed in animals are often difficult to replicate in humans. These limitations, which interact and compound each other, constitute the core obstacles preventing exosome-based therapies from moving from the laboratory to the clinic, and require gradual resolution through industry consensus, standard establishment, and multi-center collaboration.

### 6.2 Clinical translation practices in different disease areas

The progress of clinical translation of exosome-based therapies varies across different disease areas, and the following sections summarize the status by major disease type.

#### 6.2.1 Oncology

Clinical research on exosomes in tumor diagnosis and therapy is relatively active, but overall remains at the preclinical and early clinical exploratory stage. In terms of diagnosis, exosome-based liquid biopsy technologies have entered clinical validation phases. A multi-center clinical study entitled “Early Diagnosis of Lung Cancer Using Plasma-Derived Exosomes” (NCT04529915) is actively exploring exosome-based early diagnostic strategies for lung cancer (205). This study plans to enroll 470 subjects to achieve non-invasive early screening for lung cancer through detection of plasma exosomes (206). The above diagnostic studies are at the early clinical validation stage and still require large-scale prospective cohorts to confirm their clinical sensitivity and specificity.

In terms of therapy, most current strategies remain at the preclinical research stage. Exosomes combined with immune checkpoint inhibitors have demonstrated synergistic effects. For example, targeted exosomal RAB10 combined with PD-L1 inhibitors can restore M1 macrophage polarization and CD8^+^ T cell activity (129). The FD9R-GW/siIRF3 nanocomplex, which inhibits tumor-derived exosomal PD-L1 release and silences the IRF3 gene, significantly enhances antitumor immune responses when combined with anti-PD-1 immunotherapy (167). In overcoming chemotherapy resistance, targeted exosomal FABP5 combined with chemotherapy can create an immunosuppressive microenvironment by inducing M2 macrophage polarization (122). Furthermore, exosome secretion inhibitors can significantly reduce the release of tumor exosomal PD-L1 and enhance the efficacy of anti-PD-L1 antibodies. Approved drugs such as macitentan and atorvastatin also exhibit exosome secretion inhibitory activity, providing potential candidate drugs for clinical translation of exosome-targeted therapies (186). The above therapeutic strategies are all based on cell and animal models and still require entry into clinical trials to verify their efficacy and safety in humans.

#### 6.2.2 Inflammatory and immune diseases

Exosome applications in inflammatory and immune diseases have also made significant progress, with some areas having entered early-stage clinical trials. In terms of biomarkers, exosomal miR-26b-3p levels in peripheral blood of patients with Crohn’s disease are positively correlated with the degree of postoperative anastomotic inflammation (58). Exosomal miR-122-5p levels in neutrophils from patients with Behçet’s disease are negatively correlated with disease activity (144). These biomarker studies are still at the early clinical exploratory stage, and their clinical utility requires further validation in larger, multi-center cohorts.

In terms of clinical translation, a Phase II clinical trial evaluated the safety and efficacy of locally administered umbilical cord mesenchymal stem cell-derived exosomes for the treatment of refractory perianal fistulas in Crohn’s disease. This study enrolled 23 patients, with 20 completing the trial. Results showed that 12 patients achieved complete closure of all fistulas, with 30 fistulas reaching complete closure; an additional 4 patients achieved partial improvement, and no exosome-related adverse events were observed during the treatment period (207). A systematic review and single-arm meta-analysis further confirmed that local administration of mesenchymal stem cell-derived exosomes achieved complete healing in 57% of patients with complex anal fistulas and complete fistula closure in 68% of fistulas, with no systemic or local adverse reactions reported (208). In acute respiratory distress syndrome (ARDS), exosomes overexpressing CD24 (EXO-CD24) administered via aerosolized inhalation have completed a Phase IIb clinical trial enrolling 91 patients with mild-to-moderate COVID-19-associated ARDS. Results showed that 83.7% of patients experienced improvement in respiratory symptoms by day 7, 64% achieved oxygen saturation >94%, and 82.8% showed a reduction in inflammatory markers of ≥50%, with no treatment-related adverse events observed (209). The above Phase II and IIb clinical trials have preliminarily validated the safety and efficacy of exosome-based therapies in inflammatory and immune diseases; however, their therapeutic efficacy still requires further confirmation in large-scale randomized controlled trials.

#### 6.2.3 Tissue repair and regeneration

Clinical translation of exosomes in the field of tissue repair and regeneration is progressing relatively rapidly, with some directions having entered early-stage clinical trials. In neurological diseases, a clinical trial involving 18 patients demonstrated that intranasal administration of mesenchymal stem cell-derived exosomes was safe and well-tolerated, with varying degrees of muscle strength improvement and delayed disease progression observed in all but one patient (210). In osteoarthritis, clinical studies on human umbilical cord mesenchymal stem cell-derived exosomes have advanced from preclinical to clinical stages. One study systematically verified that human umbilical cord mesenchymal stem cell-derived exosomes alleviate articular cartilage inflammatory responses and promote cartilage regeneration in both *in vitro* and *in vivo* experiments, demonstrating favorable safety and efficacy in both preclinical and clinical studies (211). Systematic reviews and meta-analyses have shown that platelet-derived extracellular vesicles, which are rich in growth factors, can promote angiogenesis in diabetic wounds and alleviate inflammatory responses (212). The above studies are primarily small-sample early-phase clinical trials or preclinical studies, and their long-term efficacy and safety still require confirmation through large-sample, long-term follow-up studies.

In summary, the progress of clinical translation of exosome-based therapies varies considerably across different disease areas: the oncology field remains predominantly at the preclinical research stage, the inflammatory and immune disease field has entered Phase II validation, and the tissue repair and regeneration field has seen several early-phase clinical trials completed. Currently, no exosome-based products have been approved for marketing. Overall, the clinical translation of exosome-based therapies is still at an early stage, and data from large-scale randomized controlled trials have yet to be accumulated.

## 7 Concluding remarks

In recent years, the critical roles of exosomes in intercellular communication and immune regulation have attracted increasing attention (213). This review systematically summarizes the functional regulation of macrophages by exosomes in terms of polarization, activation, recruitment, and programmed cell death. We categorize the signaling pathways into two major groups: conservative core pathways represented by PI3K/AKT, STAT3, and NF-κB, and context-dependent special mechanisms represented by MAPK and PTEN. Furthermore, we elucidate the key roles of non-coding RNAs and metabolic reprogramming in exosome-mediated regulation of macrophage functional differentiation.

Studies have shown that exosomes from different sources exhibit significant heterogeneity in their core cargo molecules, signal transduction modes, and regulatory effects (214). Tumor-derived exosomes generally promote immune evasion and tumor progression by inducing M2 polarization, although some can induce M1 polarization and inhibit tumors. Stem cell-derived exosomes predominantly exert anti-inflammatory and tissue-repairing effects by inducing M2 polarization. Macrophage uptake of exosomes is highly selective and context-dependent, and the uptake efficiency, together with cargo properties, jointly determines the functional outcome.

Despite considerable progress, this field still faces multiple bottlenecks at both the methodological and clinical translation levels. At the level of heterogeneity resolution, the vast majority of studies are based on bulk sample analyses, and the results reflect average signals from vesicle populations, making it difficult to reveal differences in molecular composition and functional potential among individual exosomes. At the methodological level, there is a lack of standardized protocols for exosome isolation, purification, characterization, and nomenclature reporting. Products obtained through different methods differ significantly in purity and integrity. Moreover, most studies broadly refer to sEV-enriched products as “exosomes” without rigorously verifying their endosomal origin, severely constraining cross-study comparison and integration. At the clinical translation level, the vast majority of findings remain at the stage of cell lines or animal models, with relatively few validations using primary human cells, and only a handful have entered clinical trials. Even among those that have, common issues include small sample sizes and lack of randomized controls.

Given the above bottlenecks, future research should focus on the following directions. At the basic mechanistic level, single-vesicle analysis technologies should be developed to elucidate the correspondence between molecular heterogeneity and functional differences at the single-vesicle level. In parallel, spatial multi-omics approaches should be introduced to map the spatial interaction landscape between exosomes and macrophage subsets *in situ* within tissues. At the methodological level, the MISEV2023 guidelines should be fully adopted to establish a standardized framework for vesicle characterization and quality control, enhancing cross-study comparability and reproducibility. At the clinical translation level, large-scale, multi-center prospective clinical validation of exosome-based liquid biopsy biomarkers should be promoted to define their practical value in early diagnosis, subtype classification, and therapeutic response prediction. Meanwhile, scalable production processes compliant with GMP standards should be established to accelerate the transition of exosome-based therapies from basic research to clinical application. Only through coordinated advancement across these three dimensions—basic mechanistic elucidation, methodological standardization, and clinical validation—can the translation of exosome-mediated macrophage regulation research from laboratory discoveries to clinical benefits be truly achieved.
